# Hemoglobin During Pregnancy Does Not Mediate the Relationship between Nutrition Supplements and Intrauterine Growth: A Secondary Data Analysis of Women First Preconception Nutrition Trial

**DOI:** 10.1016/j.tjnut.2025.04.036

**Published:** 2025-05-19

**Authors:** Sumera Aziz Ali, Linda Valeri, Ka Kahe, Jeanine M Genkinger, Sarah Saleem, Saleem Jessani, Robert L Goldenberg, Jamie E Westcott, Jennifer F Kemp, Ana L Garcés, Lester Figueroa, Shivaprasad S Goudar, Sangappa M Dhaded, Richard J Derman, Antoinette Tshefu, Adrien L Lokangaka, Melissa S Bauserman, Elizabeth M McClure, Marion Koso Thomas, Louise Kuhn, Nancy F Krebs, Women First Preconception Nutrition Trial Group, Omrana Pasha Razzak, Umber Khan, Manjunath Somannavar, Juile M Long, Vanessa R Thorsten, Abhik Das, Audrey E Hendricks

**Affiliations:** 1 Department of Pediatrics, University of Alberta, Edmonton, AB, Canada; 2 Department of Biostatistics, Mailman School of Public Health, Columbia University Irving Medical Center, New York, NY, United States; 3 Department of Epidemiology, Harvard T.H. Chan School of Public Health, Boston, MA, United States; 4 Department of Epidemiology, Mailman School of Public Health, Columbia University Irving Medical Center, New York, NY, United States; 5 Department of Obstetrics/Gynecology, Columbia University Irving Medical Center, New York, NY, United States; 6 Herbert Irving Comprehensive Cancer Center, Columbia University Irving Medical Center, New York, NY, United States; 7 Department of Community Health Sciences, Aga Khan University, Karachi, Pakistan; 8 Department of Pediatrics, Section of Nutrition, University of Colorado Denver Anschutz Medical Campus, Aurora, CO, United States; 9 Institute of Nutrition in Central America and Panama, Guatemala City, Guatemala; 10 KLE Academy of Higher Education and Research’s Jawaharlal Nehru Medical College, Belagavi, India; 11 Thomas Jefferson University, Philadelphia, PA, United States; 12 Kinshasa School of Public Health, Kinshasa, Democratic Republic of the Congo; 13 Department of Pediatrics, University of North Carolina at Chapel Hill, Chapel Hill, NC, United States; 14 RTI International, Durham, NC, United States; 15 Eunice Kennedy Shriver National Institute of Child Health and Human Development, Bethesda, MD, United States; 16 Gertrude H. Sergievsky Center, Vagelos College of Physicians and Surgeons, Columbia University Irving Medical Center, New York, NY, United States

**Keywords:** lipid-based nutrient supplement, hemoglobin during pregnancy, intrauterine growth, causal mediation analysis, Women First Trial

## Abstract

**Background:**

Nutrition supplements such as multiple micronutrient-fortified small-quantity lipid-based nutrient supplementation (SQ-LNS) consumed either before or during pregnancy have been shown to improve intrauterine growth, but the mechanisms through which the supplements improve intrauterine growth remain unclear.

**Objectives:**

We examined whether hemoglobin (Hb) during pregnancy could be a potential mechanism through which multiple micronutrient-fortified SQ-LNS improve intrauterine growth.

**Methods:**

We used data collected from women and newborns in a randomized controlled trial conducted in Pakistan, India, the Democratic Republic of the Congo, and Guatemala. Women were randomly assigned to consume multiple micronutrient-fortified SQ-LNS from preconception until birth (arm 1); consume the SQ-LNS from the second trimester of pregnancy until birth (arm 2); or no supplement (arm 3). Intrauterine growth, expressed as birth length, weight, and head circumference *Z*-scores, was the outcome. The mediator was Hb (g/dL) measured at 12 (*n =* 2075) and 32 wk of gestation (*n =* 2157). Causal mediation analysis was employed to estimate direct and indirect effects.

**Results:**

Hb levels at 12 or 32 wk of gestation did not mediate the relation between the SQ-LNS and intrauterine growth. Indirect effects of preconception SQ-LNS (arm 1) compared with arm 3, mediated by Hb at 12 wk of gestation, were 0.02 [95% confidence interval (CI): –0.02, 0.01], 0.01 (95% CI: –0.01, 0.02), and 0.01 (95% CI: –0.01, 0.02) for length, weight, and head circumference *Z*-scores, respectively. The corresponding direct effects (95% CIs), not mediated by Hb, were 0.18 (0.09, 0.33), 0.12 (0.03, 0.23), and 0.06 (–0.03, 0.20), respectively. Site-specific and gestational age-adjusted data analyses at 12 and 32 wk of gestation confirmed the findings of no statistically significant mediated effects of Hb during pregnancy.

**Conclusions:**

The observed main effect of multiple micronutrient-fortified SQ-LNS on intrauterine growth was not mediated by Hb levels at 12 or 32 wk of gestation. The findings suggest exploring other pathways implicated in the association between the SQ-LNS and intrauterine growth.

This trial was registered at clinicaltrials.gov as #NCT01883193 (https://clinicaltrials.gov/ct2/show/NCT01883193?term=01883193&rank=1).

## Introduction

Impaired intrauterine growth is a major contributor to neonatal morbidity and mortality [1,2] and is commonly assessed using birth weight, length, and head circumference [3]. The burden of impaired intrauterine growth is particularly high in resource-limited settings [4]. Findings from a multicountry women first (WF) trial highlight significant deficits in birth size across resource-poor settings [Guatemala, Democratic Republic of Congo (DRC), India, and Pakistan] [5]. Specifically, 14% of newborns were severely short at birth, with prevalence ranging from 7.5% in Guatemala to 18.4% in Pakistan [5]. Low birth weight was observed in 27.2% of newborns, with site-specific rates varying from 14.4% in Guatemala to 34.8% in India [5]. Wasting was more prevalent, affecting 34.1% overall, with the highest rate in India (54.4%) [5]. Small head size was observed in 7.6% of newborns, with the highest prevalence in Pakistan (12.1%) [5].

The etiology of impaired intrauterine growth is multifactorial, with maternal nutrition playing a central role [6,7]. Although nutritional interventions such as iron and folic acid, multiple micronutrients, and lipid-based nutrient supplements have demonstrated benefits, their effects remain variable [[8], [9], [10], [11], [12]]. The WF trial demonstrated that multiple micronutrient-fortified small-quantity lipid-based nutrient supplementation (SQ-LNS), particularly when initiated before conception or in early pregnancy, significantly improves birth size outcomes [5]. Supplementation was associated with greater birth length and weight as well as lower rates of stunting, wasting, and underweight at birth [5]. Secondary analyses of the WF trial further suggest that multiple micronutrient-fortified SQ-LNS reduces fetal growth deficits, particularly in South Asian populations [13] and among anemic or nulliparous women [14].

Despite these findings, the biological mechanisms through which nutrient supplementation, such as SQ-LNS, influences intrauterine growth remain unclear [15]. Identifying these pathways could strengthen the biological plausibility of the SQ-LNS benefits for fetal growth. One plausible mechanism is through the reduction of maternal anemia. Studies indicate that nutritional supplementation, including iron, folic acid, and multiple micronutrients, decreases anemia prevalence during pregnancy by 31%–79%, reinforcing a causal link between supplementation and anemia prevention [10,12,16,17]. Anemia during pregnancy has consistently been associated with adverse birth outcomes, including low birth weight, small head circumference, and short birth length [[18], [19], [20], [21]]. Anemic women are more likely to give birth to infants with compromised growth compared with nonanemic women [18,19,22]. These findings suggest that multiple micronutrient-fortified SQ-LNS may improve intrauterine growth by mitigating maternal anemia.

However, prior studies have not directly examined hemoglobin (Hb) during pregnancy as a mediating pathway for fetal growth improvements. Leveraging data from the WF trial [5], we aimed to investigate whether Hb concentrations during pregnancy mediate the effect of the SQ-LNS on birth weight, length, and head circumference. We hypothesized that the benefits of multiple micronutrient-fortified SQ-LNS on intrauterine growth are at least partially mediated through improved maternal Hb levels.

Hb was selected as the mediator for several reasons. First, prior research supports its role in linking nutrition supplementation to improved fetal growth outcomes [10,12,[16], [17], [18], [19], [20], [21], [22]]. Second, Hb data were consistently available across all WF trial sites, whereas other nutrient biomarkers were not uniformly collected in the DRC or for the control arm in India. Third, gestational weight gain (GWG) was not considered a mediator, as Bauserman et al. [23] previously analyzed its role in the WF trial and found no mediating effect. Therefore, we sought to determine whether Hb levels mediate the relationship between multiple micronutrient-fortified SQ-LNS and intrauterine growth in the WF trial.

## Methods

### Study design, setting, and study population

We performed a secondary data analysis of the WF Preconception Maternal Nutrition Trial, an individual randomized controlled trial with 3 arms [5,24]. Women randomly assigned to arm 1 consumed multiple micronutrient-fortified SQ-LNS daily for ≥3 mo before conception and continued until birth. Women in arm 2 consumed the SQ-LNS from the second trimester of pregnancy until birth. Women in arm 3 did not consume the SQ-LNS (control arm) [24]. Women in the trial, enrolled from Pakistan, India, DRC, and Guatemala, were 16–35 y old with a baseline Hb level of ≥8 g/dL and were planning to conceive during the subsequent 18 mo [5,24]. Details and main results of the trial are previously published [5].

### Randomization and blinding

The Data Coordinating Center (DCC) was responsible for creating the randomization scheme and the allocation sequence for each site [5,24]. To ensure geographic balance, the DCC employed stratified randomization by geographic clusters. Within each cluster, block randomization was used to assign participants to 1 of the 3 trial arms in a 1:1:1 ratio [5,24]. The block sizes were randomly chosen from 3, 6, or 9 at each site. Once an eligible participant was identified by the responsible home visitor research assistant, the site-specific data manager generated the random assignment through the centralized computerized data management system maintained by the DCC. Although participants and research investigators were aware of the assigned intervention, the assessment team, responsible for measuring the outcomes, was blinded to the intervention assignments [5,24].

### Ethical considerations

The study was approved by the Colorado Multiple Institutional Review Board at the University of Colorado and the local and/or national ethics committees for each of the 4 sites (Guatemala, India, DRC, and Pakistan). Written informed consent was obtained from all study participants. The study protocol, available online https://www.ncbi.nlm.nih.gov/pmc/articles/PMC4000057/ [24], ensured that a data monitoring committee designated by the Eunice Kennedy Shriver National Institute of Child Health and Human Development oversaw the trial's safety and monitored adverse events.

### Intervention and outcome

The intervention was a comprehensive multiple micronutrient-fortified SQ-LNS comprising iron, folic acid, vitamins, iodine, copper, calcium, phosphorus, magnesium, potassium, selenium, zinc, proteins, polyunsaturated fatty acids, and monoglycerides (118 kcal per 20g) [24]. Three markers, newborn’s weight, length, and head circumference, measured within 48 h of birth, were used as proxy measures for intrauterine growth, the outcome of the current analysis [5,24]. More precisely, the outcome measures were *Z*-scores for birth length, weight, and head circumference based on the WHO child growth standards that account for the newborn’s sex and chronological age [25]. For India, Pakistan, and Guatemala, gestational age (GA) data were obtained by measuring fetal crown-rump length using ultrasound. We performed additional analysis using the *Z*-scores based on INTERGROWTH-21st international fetal growth standards that accounted for the GA and newborn’s sex [26].

### Mediator and analytical sample

Hb level during pregnancy was a hypothesized mediator (M) between multiple micronutrient-fortified SQ-LNS (X: intervention) and the 3 markers of intrauterine growth (Y: outcome). In the WF trial, a woman's blood sample was obtained from a finger stick to measure Hb (g/dL) at 10–12 and 32–34 wk of gestation. We used calibrated HemoCue devices (HemoCue Hb 201^+^ System; HemoCue America, Brea, CA 92821) to measure women’s Hb in Guatemala, Pakistan, and DRC [27]. For women in India, Hb was measured using Sahli’s method [28]. The analytical sample included women–newborn dyads with complete data on Hb during pregnancy and birth weight, length, and head circumference.

### Statistical analyses

#### Primary mediation analysis

Causal mediation analysis was employed to decompose the main effect of the multiple micronutrient-fortified SQ-LNS (X: intervention) on intrauterine growth (Y: outcome) into the indirect effect relative to the direct effect through pathways independent of Hb during pregnancy (M: mediator). We employed multivariable linear regression models using a counterfactual approach to mediation analysis [29]. Specifically, we first modeled the effect of the multiple micronutrient-fortified SQ-LNS on Hb levels during pregnancy:

$$M_{i}=\beta_{0}+\beta_{1}\;X_{i}+\beta_{2}\;C_{i}+\epsilon_{i}$$

where *M*_*i*_ denotes Hb during pregnancy (mediator), *X*_*i*_ is the intervention (arm 1 or arm 2 compared with arm 3), *C*_*i*_ represents a vector of mediator-outcome confounders, including maternal age, education, parity, baseline Hb, prepregnancy BMI, and socioeconomic status (SES), and *ϵ*_*i*_ denotes error term.

Next, we modeled the effect of the multiple micronutrient-fortified SQ-LNS and Hb levels on intrauterine growth:

$$Y_{i}=\beta_{0}+\beta_{1}\;X_{i}+\beta_{2}\;M_{i}+\beta_{3}\;C_{i}+\epsilon_{i}$$

Because arm 2 started the multiple micronutrient-fortified SQ-LNS at 12 wk of gestation, arm 2 did not differ from arm 3 until 12 wk. Hence, we compared arm 1 (preconception) with arm 3 (control) at 12 wk and did not compared arm 2 with arm 3 at 12 wk. However, at 32 wk, both arm 1 (preconception) and arm 2 (during pregnancy) were the intervention arms; therefore, we compared arm 1 (preconception) with arm 3 (control) and arm 2 (during pregnancy) compared with arm 3 (control) at 32 wk of gestation. As previously published [5], arm 1 did not differ from arm 2; hence, we did not decompose the total effect of arm 1 compared with arm 2.

In the WF trial, we collected data on Hb both at 10–12 and 32–34 wk of gestation; therefore, we analyzed data separately at 10–12 and 32–34 wk of gestation. Stratified analysis by GA at 10–12 and 32–34 wk of gestation allowed us to assess if Hb levels at either or both durations of gestation could mediate the relationship between the SQ-LNS and intrauterine growth. Additionally, GA was not a mediator, because nutrition supplements do not affect GA [5,[30], [31], [32], [33]]. Hence, we performed additional analyses after adjusting for GA. We used the “CMAverse package” [34] in R to perform causal mediation analysis.

### Assumptions to estimate valid direct and indirect effects

Four unmeasured confounding assumptions need to be satisfied to obtain valid estimates of natural direct and indirect effects. The assumptions [29] include *1*) no unmeasured X–Y confounding: satisfied through randomization; *2*) no unmeasured M–Y confounding: addressed by adjusting for potential M–Y confounders; *3*) no unmeasured X–M confounding: addressed through randomization, and *4*) no confounding of the M–Y relationship by a descendant of X, that is, a confounder of the M–Y relationship should not be caused by X. Because confounders of the M-Y relationship (for example, age, parity, education, BMI, SES, etc.) cannot be caused by the multiple micronutrient-fortified SQ-LNS (X), violation of the fourth assumption is less likely.

### Sensitivity analyses

#### Complex models

We found an interaction between arm and maternal Hb levels at preconception and during pregnancy. To assess whether our primary analysis findings remain robust after adding interaction terms to the original model, we included 2 interaction terms, Arm∗Hb_baseline and Arm∗Hb during pregnancy, and decomposed the total effect of the SQ-LNS (X) on fetal growth (Y) into 4 components [35]: *1*) controlled direct effect (CDE), the effect of X on Y that is neither due to mediation nor due to interaction; *2*) reference interaction (Int_ref_), the effect of X on Y that is due to interaction but not mediation; *3*) mediated interaction (Int_med_), the effect of X on Y that is both due to mediation and interaction; *4*) pure indirect effect (PIE), the effect of X on Y that is purely due to mediation but not interaction. CDE and Int_ref_ were combined to estimate the direct effect, whereas Int_med_ and PIE were added to estimate the indirect effect.

The equation included the interaction terms as follows:

$$Y_{i}=\beta_{0}+\beta_{1}\;X_{i}+\beta_{2}\;M_{\text{baseline},i}+\beta_{3}\;M_{\text{pregnancy},i}+\beta_{4}\;\left(X_{i}\times M_{\text{baseline},i}\right)+\beta_{5}\;\left(X_{i}\times M_{\text{pregnancy},i}\right)+\beta_{6}\;C_{i}+\epsilon_{i}$$

where *M*_baseline_,_i_ represents Hb at baseline (prepregnancy), and *M*_pregnancy_,_i_ is Hb measured during pregnancy (at 12 and 32 wk).

To explore potential nonlinear effects of Hb, we centered Hb around its mean by subtracting the mean Hb from the raw Hb values at 10–12 and 32–34 wk of gestation and repeated the analyses with Hb included in both linear and quadratic forms. We then fit both linear and quadratic functions to the Hb values at 10–12 and 32–34 wk of gestation in our analyses. The inclusion of quadratic terms allowed us to capture any potential nonlinear relationships between Hb and intrauterine growth markers. The resulting equation was:

$$Y_{i}=\beta_{0}+\beta_{1}\;X_{i}+\beta_{2}\;M_{i\text{centered}}+\beta_{3}\;{\left(M_{i\text{centered}}\right)}^{2}+\beta_{4}\;C_{i}+\epsilon_{i}$$

where *M*_*i*centered_ is the mean-centered Hb level during pregnancy and (*M*_*i*centered_)^2^ represents the quadratic term, which allowed us to assess potential nonlinear effects of Hb during pregnancy on intrauterine growth.

### Pooling site-specific results

Evidence suggests that the 4 participating sites in the WF trial exhibit significant heterogeneity, including differences in diet, culture, and other contextual factors [36]. Therefore, to account for these variations, we pooled site-specific direct and indirect effects using a metaanalytic approach proposed for mediation analysis [37]. Specifically, for each site, the direct and indirect effects were estimated using the following equations:

$$Y_{i}^{\left(s\right)}=\beta_{0}^{\left(s\right)}+\beta_{1}^{\left(s\right)}\;X_{i}^{\left(s\right)}+\beta_{2}^{\left(s\right)}\;M_{i}^{\left(s\right)}+\beta_{3}\;C_{i}^{\left(s\right)}+\epsilon_{i}^{\left(s\right)}$$

where *s* denotes the site*, Y*_*i*_^(s)^ is the outcome (birth weight, length, and head circumference), *X*_*i*_^(s)^ is the intervention arm, and *M*_*i*_^(s)^ represents Hb levels during pregnancy for each site. The covariate vector *C*_*i*_^(s)^ includes maternal characteristics such as age, education, parity, baseline Hb, prepregnancy BMI, and SES for each site. To obtain pooled estimates across sites, random-effect models were applied to the site-specific direct and indirect effects:

$$\hat{\theta }\;pooled=\frac{\sum_{s=1}^{S}\omega s\hat{\theta }s}{\sum_{s=1}^{S}\omega s}$$

where $\hat{\theta }s$ is the site-specific estimate, and *ω*_s_ denotes the inverse variance weights for each site.

This approach allowed us to account for between-site heterogeneity and provide more robust estimates of direct and indirect effects.

### Combining intervention arms to gain power

Given the limited statistical power resulting from the smaller sample sizes in certain intervention arms, we combined the intervention arms to enhance statistical power and assess whether the findings of the primary mediation analysis vary after gaining more power. Arms 2 and 3 were similar until 12 wk of gestation; thus, we combined arms 3 and 2 at 12 wk and compared the combined arm 2+3 against arm 1. Because there was no significant difference in the effect sizes for birth weight, length, and head circumference between arm 1 and arm 2 at 32 wk of gestation [5], we combined these arms and compared the combined arm 1+2 against arm 3. The models used to assess these comparisons were as follows:

$$Y_{i}=\beta_{0}+\beta_{1}\;X_{2+3,i}+\beta_{2}\;M_{i}+\beta_{3}\;C_{i}+\epsilon_{i}$$

where *X*_2+3,*i*_ represents the combined arm 2 and arm 3 at 12 wk. For 32 wk, the model was modified as:

$$Y_{i}=\beta_{0}+\beta_{1}\;X_{1+2,i}+\beta_{2}\;M_{i}+\beta_{3}\;C_{i}+\epsilon_{i}$$

where *X*_1+2,i_ indicates the combined arm 1 and arm 2 compared against arm 3.

Combining intervention arms allowed us to increase statistical power and obtain more stable effect estimates for the direct and indirect effects.

### Complete case compared with multiple imputed data analysis

To address potential biases due to missing Hb data (15% at 12 wk and 12% at 32 wk), we compared the results of a complete case analysis with those from a multiple imputation analysis. This approach allowed us to assess the sensitivity of the estimated direct and indirect effects to the missingness of the mediator (Hb) and determine the robustness of our findings. We used multiple imputations by the chained equations method [34], which generated multiple imputed datasets based on observed data. For each imputed dataset, the mediator (*M*_*i*_^(m)^) is imputed from a model that includes observed values of the mediator, the treatment arm *X*_i_, the outcome *Y*_i_, and confounders:

$$M_{i}^{\left(m\right)}=f\left({M_{i}^{\text{obs}}}_{,}\;X_{i,}\;Y_{i,}\;\text{confounders}\right)$$

Once imputed, we fit the mediation model for each dataset:

$$Y_{i}=\beta_{0}+\beta_{1}\;X_{i}+\beta_{2}\;M_{i}^{\left(m\right)}+\epsilon_{i}$$

The total effect of the intervention *X*_i_ on the outcome *Y*_i_ is decomposed into the direct effect (DE_m_) and the indirect effect (IE_m_): TE_m_ = DE_m_ + IE_m_.

Finally, results from all imputed datasets are pooled to obtain the overall estimates of the direct and indirect effects, using Rubin’s rule [38]:

$$\hat{\theta }final=\frac{1}{M}\sum_{m=1}^{M}\hat{\theta }m$$

where $\hat{\theta }$ is the estimate from the mth imputed dataset, and *M* is the total number of imputations.

## Results

### Study participants’ flow

In the WF trial, 12,551 women were screened, with 61.2% (7686) found eligible (Supplemental Figure 1). Of these, 96.1% (7387) were randomly assigned after providing written informed consent. Among the randomly assigned women, 56% (4136) discontinued participation before pregnancy, whereas 44% (3251) conceived within 3–18 mo and were followed for birth outcomes. Birth outcome data were recorded for 98% (3188) of the pregnant women, including 519 miscarriages and 84 stillbirths. Measurements for birth weight, length, and head circumference were taken within 48 h for 2443 singleton live births. Although the outcome data were available for 2443 live births, Hb data at 12 wk were missing for 15% (*n =* 368) and at 32 wk of gestation for 12% (*n =* 286) of women with live births. Hence, the sample size for the mediation analysis at 10–12 and 32–34 wk was reduced to 2075 and 2157, respectively (Supplemental Figure 1). The flow of study participants by arm with losses and reasons for exclusion is previously published by the Women First Study Group [5].

### Characteristics of the study sample by site and intervention arm

Overall, ∼20% of women were younger than 20 y old and nulliparous (Supplemental Table 1). About one-third of women (32.3%) were not educated, 23.6% of women had a BMI < 18.5kg/m^2^, and 57.0% were anemic (Hb < 12 g/dL) at baseline. A relatively higher proportion of nulliparous women was in Pakistan (25.8%) and India (26.4%) than in the DRC (21.7%) and Guatemala (6.4%). A higher percentage of Pakistani (37.3%) and Indian women (35.7%) had BMI < 18.5 kg/m^2^ compared with Guatemala (1.3%) and DRC (17.6%). As shown in Supplemental Table 1, Pakistan had the highest percentage of women with no formal education (84.5%), whereas India had the greatest proportion of anemic women (91.5%). There were no differences in baseline characteristics by arm except for parity and maternal education (Supplemental Table 2). A greater proportion of women with no formal education was found in arm 1 (35.0%) than in arm 2 (29.7%) and arm 3 (32.2%). Similarly, arm 1 had a relatively higher percentage of nulliparous women (23.7%) than arm 2 (19.7%) and arm 3 (17.6%). The mean Hb concentrations at 12 and 32 wk of gestation were 11.2 g/dL and 10.8 g/dL, respectively. The distribution of Hb levels at both time points, stratified by study site and intervention arm, is presented in Supplemental Tables 3a and 3b.

### Relationship between the SQ-LNS (X) and Hb levels during pregnancy (M): X-M association

**FIGURE 1:**
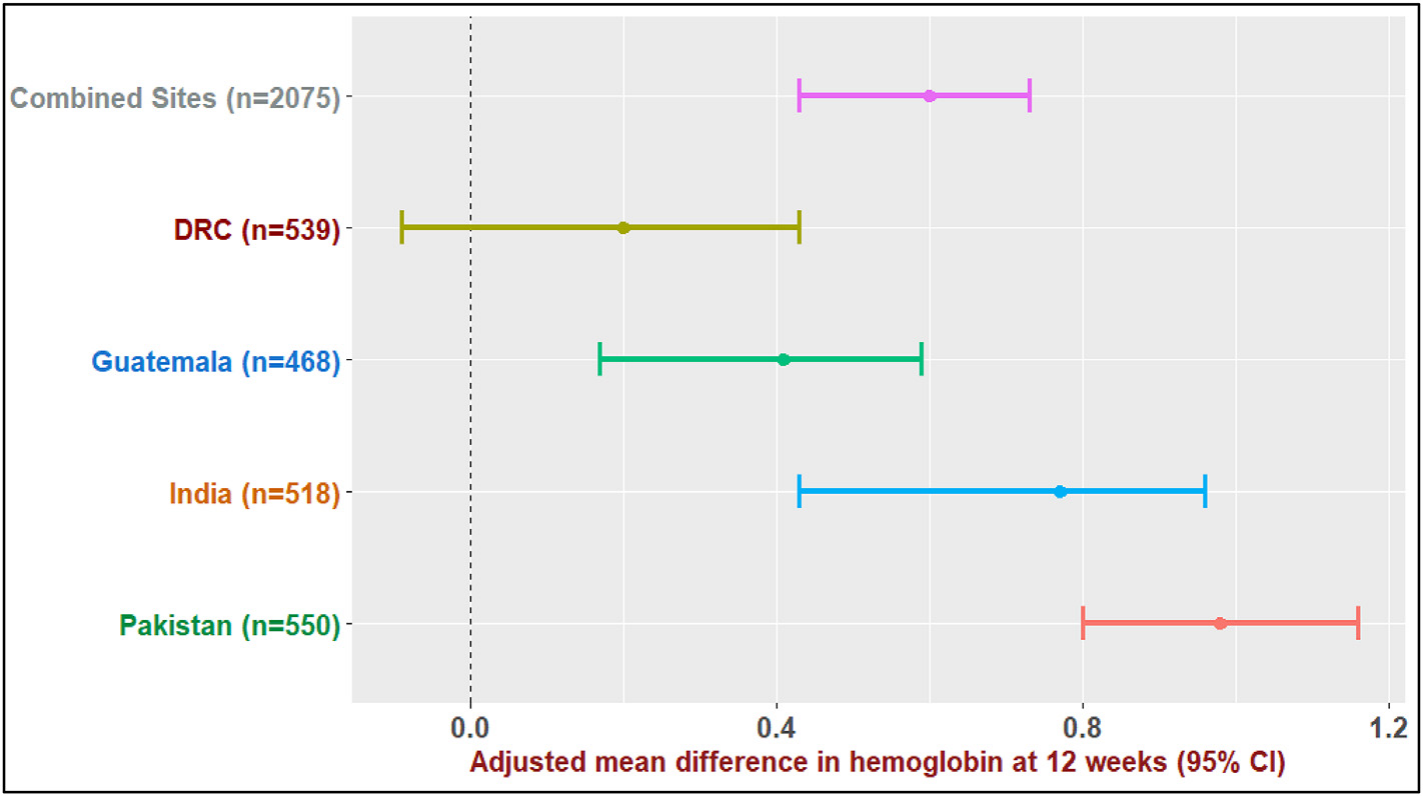
Effect of preconception small quantity lipid-based nutrient supplement (SQ-LNS) on hemoglobin (g/dL) at 12 wk of gestation: combined and site-specific analysis for SQ-LNS (intervention: X) and hemoglobin (mediator: M) association at 12 wk of gestation. The graph represents the adjusted mean difference (*β*-coefficients) in hemoglobin (g/dL) at 12 wk of gestation between arm 1 (preconception) and arm 3 (control) and corresponding 95% CIs for adjusted mean difference in hemoglobin (g/dL) at 12 wk of gestation. CI, confidence interval; DRC, Democratic Republic of Congo.

For all sites combined, the mean Hb at 12 wk of gestation was higher for arm 1 compared with arm 3 by 0.60 g/dL [95% confidence interval (CI): 0.43, 0.73], suggesting a strong association between the preconception SQ-LNS and Hb at 12 wk (Figure 1). In DRC, Guatemala, India, and Pakistan, mean Hb (95% CI) at 12 wk was higher by 0.17 (–0.09, 0.43), 0.41 (0.17, 0.59), 0.70 (0.43, 0.96), and 0.98 g/dL (0.80, 1.16), respectively, for women in arm 1 than for women in arm 3. The pattern was similar at 32 wk of gestation, however, with a lower magnitude for the corresponding adjusted mean differences in Hb at 32 wk (Supplemental Figure 2).

### Relationship between Hb concentrations during pregnancy (M) and intrauterine growth (Y): M-Y association

**FIGURE 2:**
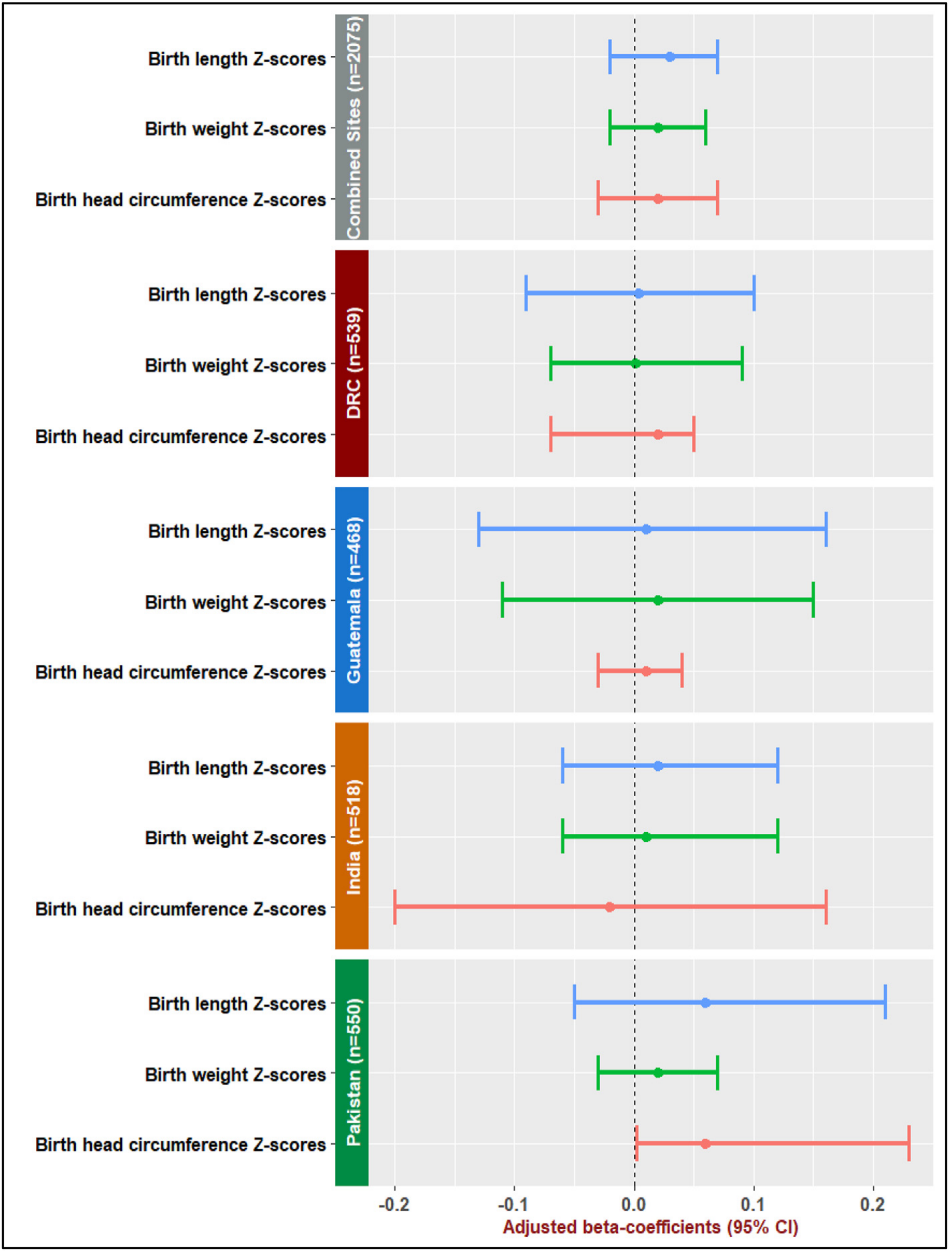
Relationship between hemoglobin (g/dL) at 12 wk of gestation (mediator: M) and 3 markers of intrauterine growth (outcome: Y): M–Y association at 12 wk of gestation. The graph represents adjusted regression coefficient for change in *Z*-scores for birth length, birth weight, and birth head circumference-for-age (Y) associated with 1 g/dL increase in hemoglobin (M) at 12 wk of gestation: Combined and site-specific analysis for M–Y association at 12 wk of gestation. M–Y confounders included in the analysis were cluster, maternal age, parity, education, socioeconomic status, prepregnancy hemoglobin, and prepregnancy BMI. CI, confidence interval

Overall, there appears to be no evidence of an association between Hb level at 12 wk of gestation (M) and intrauterine growth (Y). For 1 g/dL increment in Hb at 12 wk of gestation, the average *Z*-scores for length, weight, and head circumference-for-age at birth increased by 0.03 (95% CI: –0.02, 0.07), 0.02 (95% CI: –0.02, 0.06), and 0.02 (95% CI: –0.03, 0.07) units, respectively, adjusting for site, arm, maternal age, parity, education, SES, prepregnancy Hb, and BMI (Figure 2). Likewise, Hb at 32 wk of gestation was not associated with Z-scores for birth weight, length, and head circumference-for-age. With 1 g/dL rise in Hb at 32 wk of gestation, the average adjusted *Z*-scores for birth length, weight, and head circumference-for-age decreased by –0.01 (95% CI: –0.05, 0.02), –0.02 (95% CI: –0.03, 0.04), –0.02 (95% CI: –0.03, 0.03) units, respectively (Supplemental Figure 3).

**FIGURE 3:**
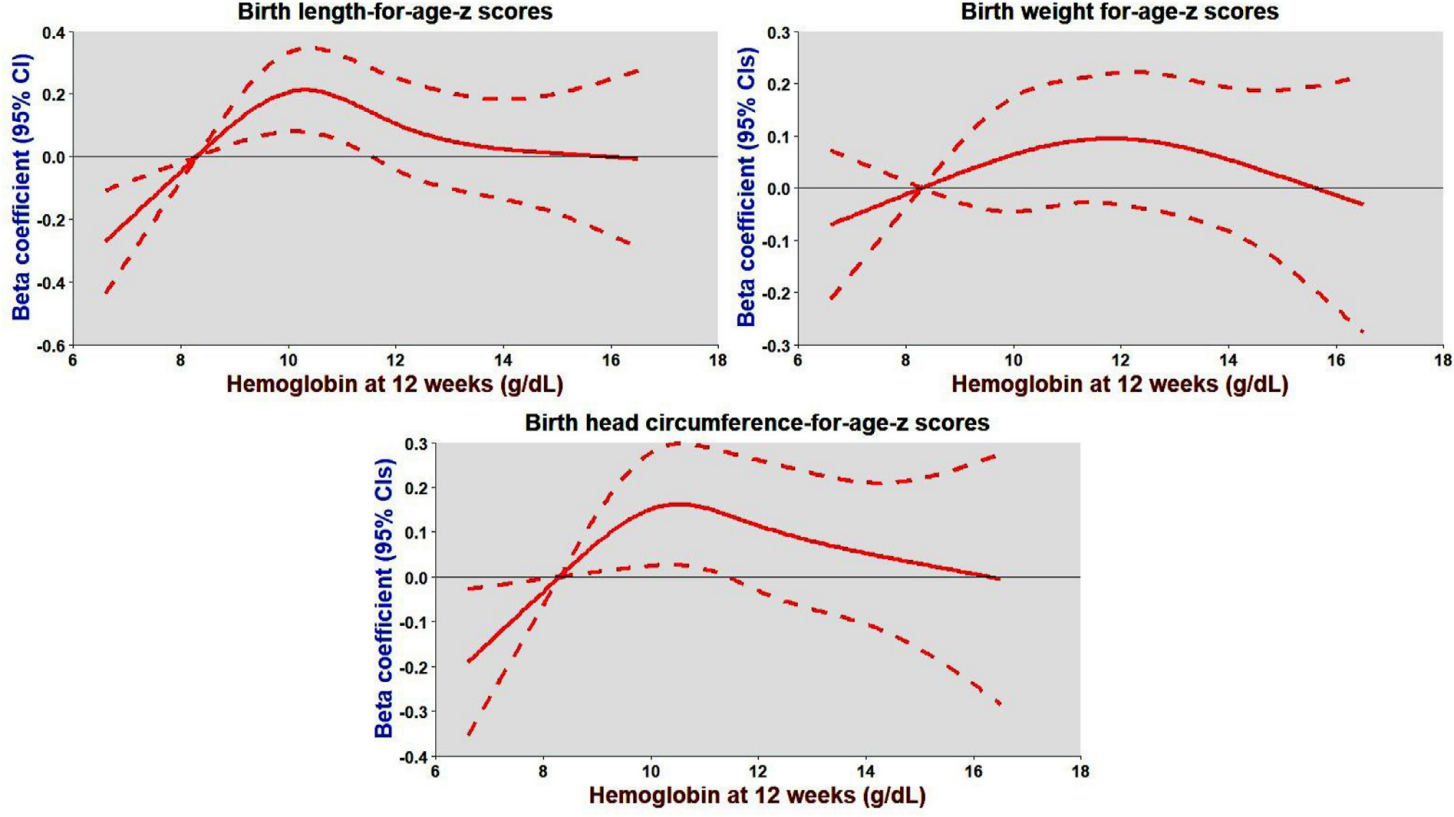
An inverted U-shaped relationship between hemoglobin (Hb) at 12 wk of gestation and 3 markers of intrauterine growth expressed as *Z*-scores for length, weight, and head circumference-for-age. Data were fitted by a restricted cubic spline regression model, and the model was fitted with 4 knots at the fifth, 35th, 65th, and 95th percentiles of Hb at 12 wk (reference is the fifth percentile). The solid red line represents beta-coefficients, that is, adjusted mean differences for change in *Z*-scores for length, weight, and head circumference associated with 1 g/dL increase in hemoglobin at 12 wk, adjusting for site, arm, maternal age, parity, education, SES, prepregnancy hemoglobin, and BMI. The shaded line represents 95% confidence intervals for the adjusted mean differences. SES, socioeconomic status.

**FIGURE 4:**
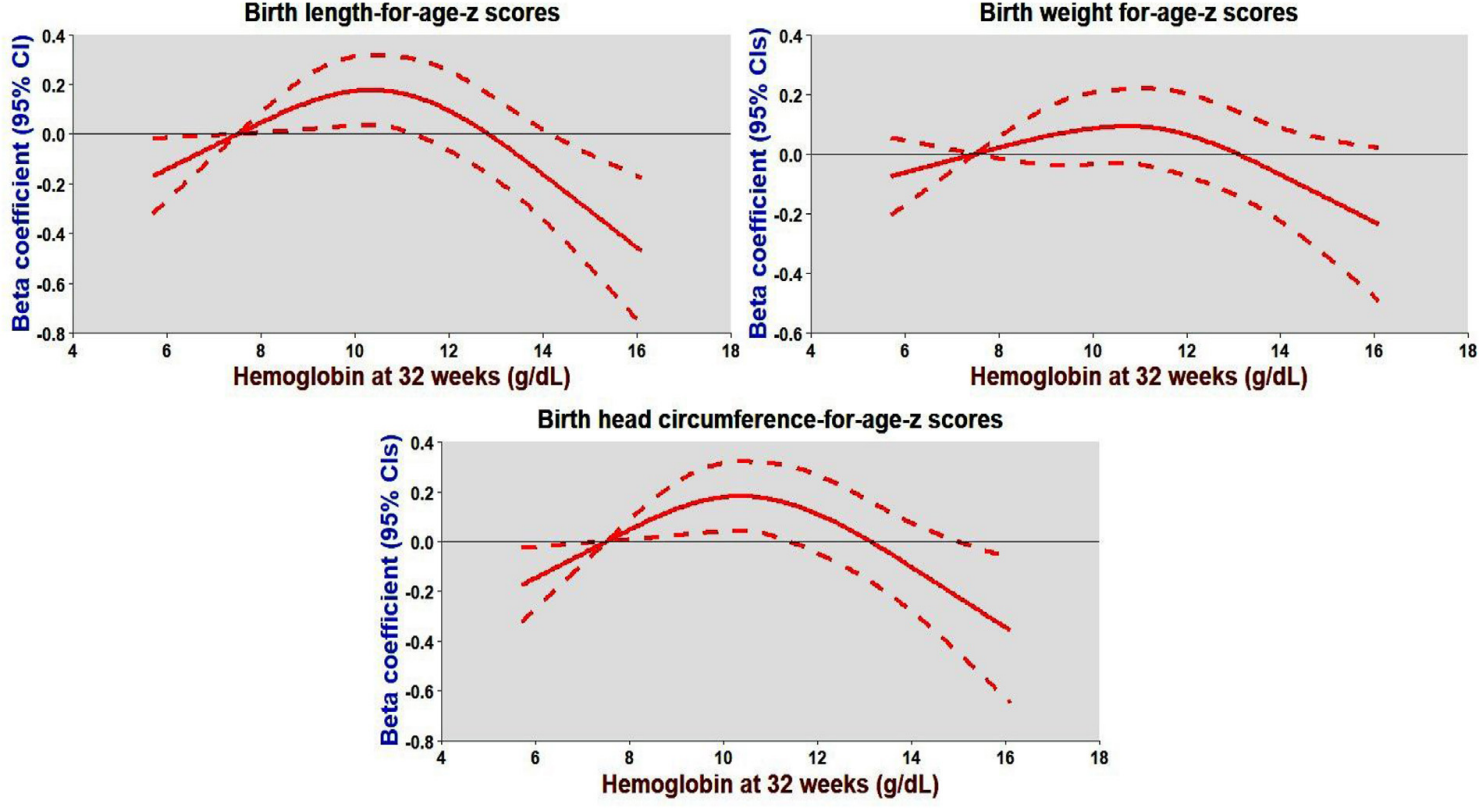
An inverted U-shaped relationship between hemoglobin (Hb) at 32 wk of gestation and 3 markers of intrauterine growth expressed as *Z*-scores for birth length, weight, and head circumference-for-age. Data were fitted by a restricted cubic spline regression model, and the model was fitted with 4 knots at the fifth, 35th, 65th, and 95th percentiles of Hb at 32 wk (reference is the fifth percentile). The solid red line represents beta-coefficients, that is, adjusted mean differences for change in *Z*-scores for birth length, weight, and head circumference-for-age associated with 1 g/dL increase in hemoglobin at 32 wk, adjusting for site, arm, maternal age, parity, education, SES, prepregnancy Hb, and BMI. The shaded line represents 95% CIs for the adjusted mean differences. CI, confidence interval; SES, socioeconomic status.

Both combined and site-specific results at 12 and 32 wk of gestation suggest no statistically significant association between Hb and intrauterine growth (M–Y association). The weak M–Y association was primarily due to the nonlinear relationship between Hb and 3 markers of fetal growth. We noticed an inverted-U-shaped relationship between Hb at 12 and 32 wk and 3 markers of intrauterine growth, suggesting women with lower and higher Hb levels (that is, extreme values) at 12 and 32 wk delivered newborns with low birth weight, short birth length, and small head circumference (FIGURE 3, FIGURE 4). This relationship is further reflected in the negative beta-coefficients for the quadratic terms of Hb at 12 and 32 wk of gestation, suggesting a nonlinear association between extreme Hb levels and poorer fetal growth outcomes.

#### Total, direct, and indirect effects of the SQ-LNS on the markers of intrauterine growth

**FIGURE 5:**
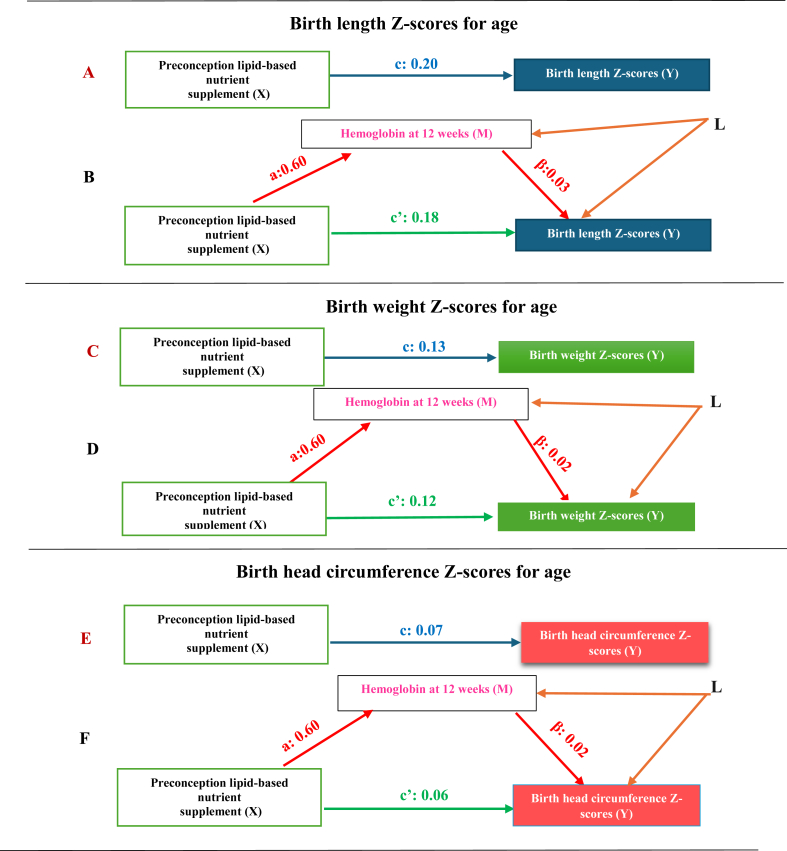
Decomposition of the total effect of preconception small quantity lipid-based nutrition supplement on 3 markers of intrauterine growth into direct and indirect effects: arm 1 (preconception) compared with arm 3 (control) at 12 wk. (A), (C), and (E) The total effect of preconception multiple micronutrient-fortified small-quantity lipid-based nutrient supplementation (SQ-LNS:X) on 3 markers of intrauterine growth, expressed as *Z*-scores of birth weight, length, and head circumference-for-age, is represented by **c**. (B), (D), and (F) Direct effect of preconception SQ-LNS (X) on 3 markers of intrauterine growth (Y) is represented by **c’** and indirect effect of SQ-LNS (X) on 3 markers of intrauterine growth (Y) through Hb at 12 wk (M) is represented by **c- c’= a∗β. a** represents the X-M association that is, the effect of preconception SQ-LNS on hemoglobin (g/dL) at 12 wk of gestation, whereas **β** represents M-Y association that is, the effect of Hb on *Z*-scores of birth weight, length, and head circumference-for-age. **L** denotes a group of measured M-Y confounders, including site, maternal age, parity, prepregnancy BMI, prepregnancy hemoglobin, socioeconomic status, and maternal education.

At 12 wk of gestation, compared with controls (arm 3), women who received the preconception SQ-LNS (arm 1) delivered newborns with higher length, more weight, and larger head circumference (Figure 5 and Supplemental Table 4). The decomposition of the total effect for arm 1 compared with arm 3 at 12 wk showed that indirect effects for birth length, weight, and head circumference-for-age *Z*-scores were 0.02 (95% CI: –0.02, 0.01), 0.01 (95% CI: –0.01, 0.02), and 0.01 (95% CI: –0.01, 0.02), respectively. The corresponding direct effects, not mediated by Hb levels at 12 wk, were 0.18 (95% CI: 0.09, 0.33), 0.12 (95% CI: 0.03, 0.23), and 0.06 (95% CI: –0.03, 0.20), respectively (Figure 5 and Supplemental Table 4). The conclusion about stronger direct and no statistically significant indirect effects persisted at 32 wk of gestation, comparing preconception SQ-LNS (arm 1) compared with controls (arm 3) (Supplemental Table 5). Similarly, arm 2 compared with arm 3 comparison at 32 wk revealed that the adjusted mean differences for direct than indirect effects were stronger for birth length (0.22; 95% CI: 0.11, 0.33), weight (0.15; 95% CI: 0.06, 0.24), and head circumference-for age-*Z*-scores (0.09; 95% CI: –0.02, 0.22) (Supplemental Table 6). Both combined and site-specific analyses (Supplemental Tables 4–6) confirmed the qualitative conclusion of no statistically significant mediated effects and substantial direct effects explained through pathways other than Hb at 12 or 32 wk of gestation, regardless of using WHO child or INTERGROWTH 21st fetal growth standards (Supplemental Table 7).

### Results of sensitivity analyses

#### Complex models

When we examined an interaction between Hb levels at 12 wk and intervention arm (Arm∗Hb_12), we found an antagonistic interaction for birth length (–0.06; *P* = 0.04), weight (–0.04; *P* = 0.11), and head circumference-for-age *Z*-scores (–0.04; *P* = 0.22). Negative signs for interaction terms imply that preconception SQ-LNS decreases length, weight, and head circumference-for-age *Z*-scores by 0.06 and 0.04 units, respectively, with a rise in Hb by 1 g/dL at 12 wk of gestation. Similarly, after keeping the interaction term between prepregnancy Hb and intervention arm (Arm∗Hb_baseline) in the model, we found significant antagonistic interactions for birth length (–0.08; *P* = 0.01), weight (–0.07; *P* = 0.01), and head circumference-for-age *Z*-scores (–0.07; *P* = 0.03). With an increment of prepregnancy Hb by 1 g/dL, the effect of preconception SQ-LNS on length, weight, and head circumference-for-age *Z*-scores declines by ∼0.08 units. However, after keeping 2 interaction terms (Arm∗Hb_12) and (Arm∗Hb_baseline) in the model simultaneously, the interaction terms became nonsignificant, albeit the negative sign persisted. The complex models with interaction terms did not change the conclusion of no mediation by Hb levels at 12 or 32 wk (Supplemental Tables 8–13).

#### Findings of additional sensitivity analysis

After comparing the combination of arms 2 and 3 compared with arm 1, the conclusion about no statistically significant mediation by Hb levels at 12 wk remained the same (Supplemental Table 14). Similarly, arm 1 + arm 2 compared with arm 3 at 32 weeks did not change the findings of no mediation by Hb levels at 32 wk (Supplemental Table 15). Given high heterogeneity (*I*^2^ = 84%), we pooled site-specific results. After pooling results for arm 1 compared with arm 3 at 12 and 32 wk of gestation, the direct effects for *Z*-scores of birth length, weight, and head circumference-for-age remained stronger than for corresponding indirect effects (Supplemental Table 16). Similarly, we did not find a substantial difference in parameter estimates after conducting multiple imputations for Hb both at 12 and 32 wk of gestation (Supplemental Table 17). The adjusted mean differences were stronger for direct than indirect effects, mediated by Hb levels. Likewise, the adjusted mean differences were stronger for direct than indirect effects, even after analyzing the sample with complete data on Hb levels both at 12 and 32 wk of gestation (*n =* 1861), as shown in Supplemental Table 18. Similarly, after performing additional analysis by dividing women into Hb <12 and ≥12 g/dL, we did not find significant mediation by Hb during pregnancy at 12 or 32 wk of gestation (Supplemental Table 19).

## Discussion

Through causal mediation analysis, we found that Hb at 12 or 32 wk of gestation does not mediate the effect of multiple micronutrient-fortified SQ-LNS, commenced either before or during pregnancy, on intrauterine growth. Rather, the entire total effect of the SQ-LNS on intrauterine growth is explained by mechanisms other than Hb levels during pregnancy. The qualitative conclusion about no statistically significant mediation by Hb at 12 or 32 wk of gestation remained the same regardless of GA adjustment, country-specific analysis, pooling findings using the metaanalytic approach, complex models with interaction terms and quadratic forms of Hb, imputing missing data for Hb, or combining intervention arms.

Compared with controls, we found a stronger effect of the SQ-LNS, especially commenced before pregnancy, on Hb levels during pregnancy, a finding consistent with previously published reviews [17,39]. However, we did not find a stronger effect of Hb levels (mediator) on fetal growth (outcome). The modest mediator-outcome relationship could be a plausible explanation for the lack of statistically significant indirect effect of the SQ-LNS on intrauterine growth. In the current analysis, Hb did not exhibit a linear relationship with fetal growth; rather, an inverted U-shaped association was observed, indicating that both low and high Hb levels (extreme Hb values) may negatively impact intrauterine growth. The inverted U-shaped relationship between Hb during pregnancy and intrauterine growth is a likely explanation for the weak mediator-outcome association and subsequent lack of statistically significant mediation by Hb levels at 12 and 32 wk of gestation. Specifically, newborns of women with Hb <9 g/dL or >11 g/dL had lower birth weight, shorter birth length, and smaller head circumference compared to those with Hb concentrations between 9 and 11 g/dL. These findings align with previous research suggesting that both anemia and hemoconcentration can impair fetal growth [[40], [41], [42], [43], [44], [45], [46], [47], [48]].

Although low Hb concentrations limit oxygen supply to the fetus [49], leading to hypoxia and potentially compromised placental perfusion [50,51], high Hb levels may indicate inadequate plasma volume expansion, a physiological adaptation critical for optimal fetal development [52,53]. During pregnancy, Hb concentrations naturally decrease due to plasma volume expansion, ensuring adequate uteroplacental blood flow [54,55]. High Hb levels, often observed in conditions like preeclampsia, can manifest from decreased intravascular volume and hemoconcentration, which impedes placental perfusion, leading to impaired fetal growth [52,53,[56], [57], [58], [59]]. Our findings support the notion that optimal intrauterine growth occurs with Hb levels between 9.5 and 11.0 g/dL during pregnancy [58,60].

To ensure the robustness of these findings, we performed additional analyses by stratifying women into 2 groups (Hb<12 g/dL and Hb ≥12 g/dL at 12 and 32 wk of GA). However, even when restricting the analysis to women with Hb concentrations below 12 g/dL, no significant mediation effect was observed. This reinforces the conclusion that Hb is not a primary mediator of the relationship between SQ-LNS and intrauterine growth, suggesting that other biological pathways may contribute to the direct effect of SQ-LNS on fetal growth.

One potential mechanism is the early improvement in maternal micronutrient status, particularly in women who received the intervention from the periconceptional period (arm 1). Micronutrient adequacy during early pregnancy may influence fetal development independently of Hb levels. However, due to data limitations, we were unable to assess the status of other nutrient biomarkers, as these measurements were not consistently available across all study sites. Furthermore, previous analyses by the WF investigators found no significant differences in GWG by treatment arm, ruling out mediation through this pathway [23]. Given the availability of data in the WF trial, we also explored BMI and GA as potential mediators. However, neither BMI nor GA showed significant mediation effects, further supporting the notion that the impact of SQ-LNS on fetal growth is not primarily driven through these pathways. Although GA and BMI were not the primary focus of this analysis, their evaluation provided additional insights into potential mechanisms that warrant further investigation.

Additional pathways through which the SQ-LNS may improve intrauterine growth warrant further exploration. One such pathway is the potential role of SQ-LNS in strengthening maternal immunity and reducing susceptibility to infections, thereby lowering risk of preterm birth and low birth weight [61]. Nutrients within SQ-LNS, particularly those involved in immune function, may contribute to a more favorable intrauterine environment [61]. Another plausible mechanism is the effect of supplementation on maternal plasma volume expansion, which is critical for optimal fetal nutrient delivery and growth [62]. Furthermore, fatty acids obtained from macronutrients in SQ-LNS may influence fetal fat deposition via higher cord blood leptin levels, which have been positively associated with birth size [63]. Although our dataset does not allow direct investigation of these pathways, future research should explore these alternative mechanisms to better understand the effects of maternal supplementation on fetal growth.

### Strengths and limitations

The current analysis is the first of its kind that ruled out Hb during pregnancy as a mediator between the SQ-LNS and intrauterine growth. We employed a counterfactual approach to causal mediation analysis to disentangle the direct and indirect effects of the SQ-LNS on intrauterine growth within a causal framework. The causal mediation analysis demonstrated how the total effect of the SQ-LNS can be partitioned into direct and indirect effects even in the presence of exposure and mediator interaction. Second, the data from the WF trial helped to establish temporality between exposure, mediator, and outcome. Temporality is critical for mediation analysis, which requires the mediator to be influenced by the intervention but not by the outcome. Temporality is guaranteed here because the SQ-LNS was assigned before conception, and mediator (Hb) and intrauterine growth were prospectively measured over time. Third, for the causal mediation analysis, it is necessary to adjust for confounders to obtain valid direct and indirect estimates. Although randomization reduces the chances of confounding between the SQ-LNS and outcome (X–Y) or SQ-LNS and mediator (X–M), the mediator-outcome (M–Y) relationship might be confounded because the mediator (Hb) was not randomly assigned. We adjusted for potential M–Y confounders to obtain less biased estimates for direct and indirect effects.

However, there are some limitations. First, multiple imputations did not allow us to completely rule out the possibility of selection bias introduced by missing Hb levels at 12 and 32 wk of gestation. However, getting similar estimates with and without imputed Hb data provided some reassurance that the findings about no statistically significant indirect effects were not entirely explained by selection bias due to missing Hb. Another limitation pertains to the unmeasured M–Y confounding because we did not measure and adjust for all potential M–Y confounders. Positive confounders such as the number of antenatal care visits, malaria infections, or dietary intake of women during pregnancy could have confounded the estimates for direct and indirect effects. Without adjusting for these positive confounders, the indirect effects would be overestimated. However, the issue of unmeasured M–Y confounders may not be relevant in the current analysis, with no statistically significant indirect effects. Rather, adjusting for the unmeasured positive confounders would have strengthened our conclusion about no indirect and substantial direct effects. One limitation of this study is the small sample size, which may have limited the power to detect significant indirect effects. As noted in the literature, mediation analysis and analyses involving interaction terms typically require larger sample sizes to achieve adequate statistical power [64]. The limited sample size may have contributed to the null findings for the mediated effect observed in this analysis.

In conclusion, causal mediation analysis reveals important insights regarding the role Hb levels during pregnancy play in the relationship between multiple micronutrient-fortified SQ-LNS and intrauterine growth. The findings suggest that Hb levels at 12 or 32 wk of gestation do not mediate the improvement of fetal growth by the SQ-LNS. Rather, the direct effect of the SQ-LNS on intrauterine growth accounts for most of the total effect, raising the important question of what these pathways might be. Further investigation of alternative mediators of the association between the SQ-LNS and intrauterine growth is warranted.

## Author contributions

The authors’ responsibilities were as follows – SAA: a site investigator for Pakistan, proposed research question and hypothesis, performed statistical analysis for the current manuscript, wrote paper and edited and reviewed the manuscript; LV: supervised the statistical analysis and edited and reviewed the manuscript; KK, JMG: supervised the work, reviewed and edited the manuscript; SS, RLG: supervised the research activities for Pakistan and edited and reviewed the manuscript; SJ: investigated and administered research project for Pakistan, edited, and reviewed the manuscript; JEW: supervised the project work for all 4 participating countries, was involved in methodology, project management, investigation, supervision, and writing and editing of the manuscript; JFK, EMM: helped in data curation, research project methodology and administration, and writing and editing of the manuscript; ALG: supervised the project administration for Guatemala, edited, and reviewed the manuscript; LF: involved in investigation and research project administration for Guatemala; SSG, RJD: supervised the research project activities for India, edited, and reviewed the manuscript; SMD: involved in investigation and project administration for India and edited and reviewed the manuscript; AT: supervised the research project administration for DRC and edited and reviewed the manuscript; ALL: involved in investigation and project administration for DRC and edited and reviewed the manuscript; MSB: supervised the research project activities for DRC and edited and reviewed the manuscript; MKT: oversaw the WF trial for all 4 countries, reviewed and edited the manuscript; LK: supervised the analysis, write up and details of the project, reviewed and edited various drafts of the manuscript; NFK: conceptualized primary WF trial, obtained funding, was involved in project methodology and management, provided resources, supervised research work for all 4 countries, and reviewed and edited the manuscript; and all authors: read and approved the final manuscript.

## Data availability

On publication of the study findings, deidentified study data will be accessible through the National Institute of Child Health and Human Development Data and Specimen Hub at https://dash.nichd.nih.gov.

## Funding

The Bill and Melinda Gates Foundation, the Eunice Kennedy Shriver National Institute of Child Health and Human Development, and the Office of Dietary Supplements funded the WF preconception maternal nutritional trial.

## Conflict of interest

SAA reports financial support was provided byFulbright scholarship and NIH Office of Dietary Supplements. JW reports financial support was provided by NIH, BMGF, NIH Office of Dietary Supplements. LV reports financial support was provided by Harvard University, University of Michigan, Erasmus Universiteit Rotterdam Statistical Horizons, LLC. LV reports a relationship with Harvard University and University of Michigan that includes: speaking and lecture fees. UK reports financial support was provided by NIH, BMGF, NIH Office of Dietary Supplements. VT reports financial support was provided by NIH, BMGF, NIH Office of Dietary Supplements. EM reports financial support was provided by NIH, BMGF, NIH Office of Dietary Supplements. AL reports financial support was provided by NIH, BMGF, NIH Office of Dietary Supplements. NFK reports financial support was provided by NIH, BMGF, NIH Office of Dietary Supplements. MKT reports was provided by NIH, BMGF, NIH Office of Dietary Supplements. JFK reports was provided by NIH, BMGF, NIH Office of Dietary Supplements. AEH reports financial support was provided by NIH, BMGF, NIH Office of Dietary Supplements. SG reports financial support was provided by NIH, BMGF, NIH Office of Dietary Supplements. RG reports financial support was provided by NIH, BMGF, NIH Office of Dietary Supplements. AG reports financial support was provided by NIH, BMGF, NIH Office of Dietary Supplements. LF reports financial support was provided by NIH, Bill and Melinda Gates Foundation, and NIH Office of Dietary Supplements. SMD reports financial support was provided by NIH, BMGF, NIH Office of Dietary Supplements. AD reports financial support was provided by NIH, BMGF, NIH Office of Dietary Supplements. MB reports financial support was provided by NIH, BMGF, NIH Office of Dietary Supplements. All other authors report no conflicts of interest.
