# Supplementary material for: Hemoglobin During Pregnancy Does Not Mediate the Relationship between Nutrition Supplements and Intrauterine Growth: A Secondary Data Analysis of Women First Preconception Nutrition Trial
Source: J Nutr. 2025 May 19;155(7):2385–97. doi: 10.1016/j.tjnut.2025.04.036 (PMC12308138; doi:10.1016/j.tjnut.2025.04.036)
Supplement: Multimedia component 1 [file mmc1.docx]

Women screened in the trial

n=12,551

Consented and randomized

n=7,387

Ineligible: 4,854

Refused: 310

Women became pregnant

n=3,251

Exited the study

n=4136

Delivery outcome obtained

n=3,188

Exited the study

n=63

Women with livebirths

n=2,585

Miscarriages, n= 519

Stillbirths, n= 84

Outcome measured

within 48 hours of birth: 2,443

Outcome data missing/measured after 48 hours of birth

n= 142

Complete data both on hemoglobin measured at 32-34 weeks plus

Outcome of interest (n=2,157)

**Analytical sample**

**(32-34 weeks of gestation)**

Hemoglobin measured during pregnancy

10-12 weeks, n= 2,373

32-34 weeks, n= 2,296

Complete data both on hemoglobin measured at 10-12 weeks of gestation plus

outcome of interest

(n=2,075)

**Analytical sample**

**(10-12 weeks of gestation)**

**Preconception**

**Pregnancy**

**Postpartum**

**Supplemental Figure 1:** Flow of study participants in the Women First Trial and sample size for mediation analysis: Flow of study participants by Arm with losses and reasons for exclusion is previously published by Women First Study Group.

| **Supplemental Table 1: Baseline characteristics of women included in the sample, by study site and gestational age** | | | | | | | | | | |  |
| --- | --- | --- | --- | --- | --- | --- | --- | --- | --- | --- | --- |
| **12 weeks of gestation (n=2075)** | | | | | | | | | | |  |
| **Characteristics** | **All (n=2075)** | | **India (n=518)** | | **Pakistan (n=550)** | | **DRC (n=539)** | | **Guatemala (n=468)** | |  |
| **Age (years), mean ± SD** | 23.2± 4.2 | | 21.8± 3.3 | | 23.9±4.1 | | 22.9± 4.6 | | 24.1± 4.4 | |  |
| **Age categories, *n (%)*** | | | | | | | | | | |  |
| < 20 Years | 430 (20.7) | | 128 (24.7) | | 95 (17.3) | | 134 (24.8) | | 73 (15.6) | |  |
| 20-24 years | 868 (41.8) | | 286 (55.2) | | 180 (32.7) | | 208 (38.6) | | 194 (41.4) | |  |
| ≥ 25 years | 777 (37.4) | | 104 (20.1) | | 275 (50.0) | | 197 (36.5) | | 201 (42.9) | |  |
| **Parity, *n (%)*** | | | | | | | | | | |  |
| 0 child (nulliparous) | 426 (20.5) | | 137 (26.4) | | 142 (25.8) | | 117 (21.7) | | 30 (6.4) | |  |
| 1 child | 649 (31.3) | | 216 (41.7) | | 132 (24.0) | | 117 (21.7) | | 184 (39.3) | |  |
| ≥ 2 children | 1000 (48.2) | | 165 (31.8) | | 276 (50.2) | | 305 (56.6) | | 254 (54.3) | |  |
| **Maternal Education, *n (%)*** | | | | | | | | | | |  |
| No formal education | 670 (32.3) | | 39 (7.5) | | 465 (84.5) | | 129 (23.9) | | 37 (7.9) | |  |
| Primary | 756 (36.4) | | 74 (14.3) | | 55 (10.0) | | 322 (59.7) | | 305 (65.2) | |  |
| Secondary or above | 649 (31.3) | | 405 (78.2) | | 30 (5.4) | | 88 (16.3) | | 126 (26.9) | |  |
| **Maternal BMI (kg/m^2^), mean ± SD** | 21.3± 3.9 | | 20.1± 3.4 | | 19.6± 2.8 | | 20.6± 2.6 | | 25.3± 4.3 | |  |
| **Maternal BMI (kg/m^2^) categories (1)**, ***n (%)*** | | | | | | | | | | |  |
| Underweight (< 18.5) | 491 (23.6) | | 185 (35.7) | | 205 (37.3) | | 95 (17.6) | | 6 (1.3) | |  |
| Healthy weight (18.5-24.9) | 1274 (61.4) | | 290 (55.9) | | 319 (58.0) | | 419 (77.7) | | 246 (52.5) | |  |
| Overweight or obese (≥ 25) | 310 (14.9) | | 43(8.3) | | 26 (4.7) | | 25 (4.6) | | 216 (46.1) | |  |
| **Tally indicators for socioeconomic status**^1^**, n (%)** | | | | | | | | | | |  |
| 0 indicator | 311(14.9) | | 0 (0.0) | | 18 (3.3) | | 293 (54.4) | | 0 (0.0) | |  |
| 1-2 indicators | 594 (28.6) | | 51 (9.8) | | 248 (45.1) | | 238 (44.2) | | 57 (12.2) | |  |
| 3-4 indicators | 816 (39.3) | | 329 (63.5) | | 199 (36.2) | | 8 (1.5) | | 280 (59.8) | |  |
| 5-6 indicators | | 354 (17.1) | | 138 (26.6) | | 85 (15.4) | | 0(0.0) | | 131 (27.9) | |
| **Baseline hemoglobin (Hb), mean ± SD** | | 11.6±1.8 | | 10.1±1.1 | | 10.9±1.6 | | 12.1±1.2 | | 13.3±1.5 | |
| **Pre-pregnancy anemia** (2)**, *n (%)*** | | | | | | | | | | |  |
| Anemia (Hb < 12 gm/dl) | 1183 (57.0) | | 474 (91.5) | | 397 (72.2) | | 244 (45.3) | | 68 (14.5) | |  |
| No anemia (Hb ≥ 12 gm/dl) | 892 (42.9) | | 44(8.5) | | 153 (27.8) | | 295 (54.7) | | 400 (85.5) | |  |
| **32 weeks of gestation (n=2157)** | | | | | | | | | | |  |
|  | **All (n=2157)** | | **India (n=570)** | | **Pakistan (n=614)** | | **DRC (n=436)** | | **Guatemala (n=537)** | |  |
| **Age (years), mean ± SD** | 23.2± 4.2 | | 21.9± 3.4 | | 23.7±4.0 | | 22.8± 4.5 | | 24.2± 4.4 | |  |
| **Age categories, *n (%)*** | | | | | | | | | | |  |
| < 20 Years | 439 (20.3) | | 141 (24.7) | | 109 (17.7) | | 107 (24.5) | | 82 (15.3) | |  |
| 20-24 years | 897 (41.6) | | 308 (54.0) | | 205 (33.4) | | 170 (38.9) | | 214 (39.8) | |  |
| ≥ 25 years | 821 (38.1) | | 121(21.2) | | 300 (48.8) | | 159 (36.5) | | 241 (44.8) | |  |
| **Parity, *n (%)*** | | | | | | | | | | |  |
| 0 child (nulliparous) | 434 (20.1) | | 144 (25.3) | | 174 (28.3) | | 83 (19.0) | | 33 (6.1) | |  |
| 1 child | 694 (32.2) | | 235 (41.2) | | 139 (22.6) | | 111 (25.4) | | 209 (38.9) | |  |
| ≥ 2 children | 1029 (47.7) | | 191 (33.5) | | 301(49.0) | | 242 (55.5) | | 295 (54.9) | |  |
| **Maternal Education, *n (%)*** | | | | | | | | | | |  |
| No formal education | 701 (32.5) | | 41(7.2) | | 520 (84.7) | | 99 (22.7) | | 41 (7.6) | |  |
| Primary | 764 (35.4) | | 90 (15.8) | | 61 (9.9) | | 255 (58.5) | | 358 (66.7) | |  |
| Secondary or above | 692 (32.1) | | 439 (77.0) | | 33 (5.4) | | 82 (18.8) | | 138 (25.7) | |  |
| **Maternal BMI (kg/m^2^), mean ± SD** | 21.4± 4.0 | | 20.0± 3.4 | | 19.6± 2.8 | | 20.6± 2.6 | | 25.3± 4.1 | |  |
| **Maternal BMI (kg/m^2^) categories** (1), ***n (%)*** | | | | | | | | | | |  |
| Underweight (< 18.5) | 513 (23.8) | | 209 (36.7) | | 221 (35.9) | | 77 (17.6) | | 6 (1.1) | |  |
| Healthy weight (18.5-24.9) | 1300 (60.3) | | 315 (55.3) | | 366 (59.6) | | 338 (77.5) | | 281 (52.3) | |  |
| Overweight or obese (≥ 25) | 344 (15.9) | | 46 (8.1) | | 27 (4.4) | | 21 (4.8) | | 250 (46.5) | |  |
| **Tally indicators for socioeconomic status**^1^**, n (%)** | | | | | | | | | | |  |
| 0 indicator | 247 (11.4) | | 0 (0.0) | | 18 (2.9) | | 229 (52.5) | | 0 (0.0) | |  |
| 1-2 indicators | 602 (27.9) | | 56 (9.8) | | 284 (46.2) | | 201(46.1) | | 61 (11.4) | |  |
| 3- 4 indicators | 916 (42.5) | | 366 (64.2) | | 224 (36.5) | | 6 (1.4) | | 320 (59.6) | |  |
| 5-6 indicators | 392 (18.2) | | 148 (25.9) | | 88 (14.3) | | 0 (0.0) | | 156 (29.0) | |  |
| **Baseline hemoglobin (Hb), mean ± SD** | 11.6±1.8 | | 10.1±1.1 | | 10.9±1.6 | | 12.1±1.2 | | 13.3±1.5 | |  |
| **Pre-pregnancy anemia** (2)**, *n (%)*** | | | | | | | | | | |  |
| Anemia (Hb < 12 gm/dl) | 1245 (57.7) | | 525 (91.9) | | 441(71.8) | | 199 (45.6) | | 80(14.9) | |  |
| No anemia (Hb ≥ 12 gm/dl) | 912 (42.3) | | 45 (7.9) | | 173 (28.2) | | 237 (54.4) | | 457 (85.1) | |  |

BMI, body mass index; DRC, Democratic Republic of Congo; Hb, Hemoglobin; SD, standard deviation

^1^The tally for socioeconomic status is based on the list of six indicators including electricity, sanitation, improved water supply, man-made flooring, improved water source, improved cooking fuels, and household assets. Women with 0-2 indicators belonged to low socioeconomic status.

| **Supplemental Table 2: Baseline characteristics of women included in the sample, by gestational age and intervention arm** | | | |
| --- | --- | --- | --- |
| **Gestational Age: 12 weeks (n=2075)** | | | |
| **Characteristics** | ***Arm 1***  ***(n=731)*** | ***Arm 2 (n=764)*** | ***Arm 3***  ***(n=580)*** |
| **Age (years), mean ± SD** | 23.1± 4.2 | 23.1±4.2 | 23.3± 4.2 |
| **Age categories, *n (%)*** | | | |
| <20 Years | 147 (20.1) | 166 (21.7) | 117 (20.2) |
| 20-24 years | 324 (44.3) | 315 (41.2) | 229 (39.5) |
| ≥ 25 years | 260 (35.6) | 283 (37.0) | 234 (40.3) |
| **Parity, *n (%)***^1^ | | | |
| 0 child (nulliparous) | 173 (23.7) | 151 (19.7) | 102 (17.6) |
| 1 child | 223 (30.5) | 239 (31.3) | 187 (32.2) |
| ≥ 2 children | 335 (45.8) | 374 (48.9) | 291 (50.2) |
| **Maternal Education, *n (%)***^1^ |  |  |  |
| No formal education | 256 (35.0) | 227 (29.7) | 187 (32.2) |
| Primary | 234 (32.0) | 290 (37.9) | 232 (40.0) |
| Secondary or above | 241 (32.9) | 247 (32.3) | 161(27.7) |
| **Maternal BMI (kg/m^2^), mean ± SD** | 21.3± 3.9 | 21.3± 4.0 | 21.4± 3.9 |
| **Maternal BMI (kg/m^2^) categories** (1)**, *n (%)*** | | | |
| Underweight (<18.5) | 181 (24.7) | 185 (24.2) | 125 (21.5) |
| Healthy weight (18.5-24.9) | 435 (59.5) | 468 (61.3) | 371(63.9) |
| Overweight or obese (≥25) | 115 (15.7) | 111 (14.5) | 84 (14.5) |
| **Tally indicators for socioeconomic status**^2^**, n (%)** | | | |
| 0 indicator | 102 (13.9) | 110 (14.4) | 99 (17.1) |
| 1-2 indicators | 221(30.2) | 212 (27.7) | 161 (27.7) |
| 3- 4 indicators | 282 (38.6) | 311 (40.7) | 223 (38.4) |
| 5-6 indicators | 126 (17.3) | 131(17.1) | 97 (16.7) |
| **Pre-pregnancy hemoglobin (Hb), mean ± SD** | 11.6±1.8 | 11.7±1.8 | 11.6±1.8 |
| **Pre-pregnancy anemia** (2)**, *n (%)*** | | | |
| Anemia (Hb < 12 g/dL) | 431(58.9) | 433 (56.7) | 319 (55.0) |
| No anemia (Hb ≥ 12 g/dL) | 300 (41.0) | 331 (43.3) | 261 (45.0) |
| **Gestational Age: 32 weeks (n=2157)** | | | |
|  | ***Arm 1 (n=702)*** | ***Arm 2 (n=757)*** | ***Arm 3***  ***(n=698)*** |
| **Age (years), mean ± SD** | 23.2± 4.2 | 23.1±4.1 | 23.3± 4.2 |
| **Age categories, *n (%)*** |  |  |  |
| <20 Years | 136 (19.4) | 166 (21.9) | 137 (19.6) |
| 20-24 years | 305 (43.4) | 314 (41.5) | 278 (39.8) |
| ≥ 25 years | 261 (37.2) | 277 (36.6) | 283 (40.5) |
| **Parity, *n (%)***^1^ |  |  |  |
| 0 child (nulliparous) | 169 (24.1) | 144(19.0) | 121 (17.3) |
| 1 child | 215 (30.6) | 246 (32.5) | 233 (33.4) |
| ≥ 2 children | 318 (45.3) | 367 (48.5) | 344 (49.3) |
| **Maternal Education, *n (%)***^1^ |  |  |  |
| No formal education | 253 (36.0) | 227 (29.9) | 221 (31.6) |
| Primary | 215 (30.6) | 277 (36.6) | 272 (38.9) |
| Secondary or above | 234 (33.3) | 253 (33.4) | 205 (29.4) |
| **Maternal BMI (kg/m^2^), mean ± SD** | 21.3± 3.9 | 21.4± 4.1 | 21.4±4.0 |
| **Maternal BMI (kg/m^2^) categories** (1), ***n (%)*** | | | |
| Underweight (<18.5) | 173 (24.6) | 177 (23.4) | 163 (23.3) |
| Healthy weight (18.5-24.9) | 417 (59.4) | 460 (60.7) | 423 (60.6) |
| Overweight or obese (≥25) | 112 (15.9) | 120 (15.8) | 112 (16.0) |
| **Tally indicators for socioeconomic status**^2^**, n (%)** | | | |
| 0 indicator | 81 (11.5) | 86 (11.4) | 80 (11.4) |
| 1-2 indicators | 213 (30.3) | 211 (27.8) | 178 (25.5) |
| 3-4 indicators | 284 (40.5) | 322 (42.5) | 310 (44.4) |
| 5-6 indicators | 124 (17.6) | 138 (18.2) | 130 (18.6) |
| **Pre-pregnancy hemoglobin (Hb), mean ± SD** | 11.5±1.8 | 11.6±1.8 | 11.6±1.9 |
| **Pre-pregnancy anemia** (2)**, *n (%)*** | | | |
| Anemia (Hb < 12 gm/dl) | 414 (58.9) | 432 (57.1) | 399 (57.2) |
| No anemia (Hb ≥ 12 gm/dl) | 288 (41.0) | 325 (42.9) | 299 (42.8) |

BMI, body mass index; DRC, Democratic Republic of Congo; Hb, Hemoglobin; SD, standard deviation

^1^There were significant differences in maternal education and parity by Arm.

^2^The tally for socioeconomic status is based on the list of six indicators including electricity, sanitation, improved water supply, man-made flooring, improved water source, improved cooking fuels, and household assets. Women with 0-2 indicators belonged to low socioeconomic status

| **Supplemental Table 3a: Distribution of hemoglobin at 12 weeks of gestation by Study Site and Intervention Arm** | | | | | | | | | |
| --- | --- | --- | --- | --- | --- | --- | --- | --- | --- |
|  | **All**  **(n=2075)** | **India**  **(n=518)** | **Pakistan**  **(n=550)** | **DRC**  **(n=539)** | **Guatemala (n=468)** | **Arm 1**  **(n=731)** | **Arm 2**  **(n=764)** | | **Arm 3**  **(n=580)** |
| **Hemoglobin (Hb: g/dL), mean ± SD** | 11.2±2.0 | 10.1±1.1 | 10.2±1.7 | 10.9±1.5 | 13.8±1.3 | 11.4±2.0 | 11.3±2.1 | | 10.9±2.1 |
| **Hemoglobin Status (Cutoff: 11 g/dL), n (%)** |  |  |  |  |  |  |  | |  |
| Anemia (Hb <11 g/dL) | 1052 (50.7) | 413 (79.7) | 355 (64.5) | 281 (52.1) | 3 (0.6) | 340 (46.5) | 378 (49.5) | 334 (57.6) | |
| No anemia (Hb ≥11g/dL) | 1023 (49.3) | 105 (20.3) | 195 (35.5) | 258 (47.9) | 465 (99.4) | 391 (53.5) | 386 (50.5) | 246 (42.4) | |
| **Anemia (WHO classification)** (2)**, n (%)** |  |  |  |  |  |  |  |  | |
| No anemia (Hb ≥11 g/dL) | 1023 (49.3) | 105 (20.3) | 195 (35.5) | 258 (47.9) | 465 (99.4) | 391 (53.5) | 386 (50.5) | 246 (42.4) | |
| Mild Anemia (Hb: 10-10.9 g/dL) | 465 (22.4) | 201 (38.8) | 109 (19.8) | 152 (28.2) | 3 (0.6) | 173 (23.7) | 168 (22.0) | 124 (21.4) | |
| Moderate anemia (Hb: 7-9.9 g/dL) | 568 (27.4) | 211 (40.7) | 230 (41.8) | 127 (23.6) | 0 (0.0) | 160 (21.9) | 205 (26.8) | 203 (35.0) | |
| Severe anemia (HB < 7 g/dL) | 19 (0.9) | 1 (0.2) | 16 (2.9) | 2 (0.4) | 0 | 7 (1.0) | 5 (0.7) | 7 (1.2) | |
| **HB cut-offs, n (%)** |  |  |  |  |  |  |  |  | |
| Hb: <7 g/dL | 19 (0.9) | 1 (0.2) | 16 (2.9) | 2 (0.4) | 0 (0.0) | 7 (1.0) | 5 (0.7) | 7 (1.2) | |
| Hb: 7-7.9 g/dL | 49 (2.4) | 5 (1.0) | 35 (6.4) | 9 (1.7) | 0 (0.0) | 4 (0.5) | 18 (2.4) | 27 (4.7) | |
| Hb: 8-8.9 g/dL | 151 (7.3) | 49 (9.5) | 73 (13.3) | 29 (5.4) | 0 (0.0) | 46 (6.3) | 49 (6.4) | 56 (9.7) | |
| Hb: 9-9.9 g/dL | 368 (17.7) | 157 (30.3) | 122 (22.2) | 89 (16.5) | 0 (0.0) | 110 (15.0) | 138 (18.1) | 120 (20.7) | |
| Hb: 10-10.9 g/dL | 465 (22.4) | 201 (38.8) | 109 (19.8) | 152 (28.2) | 3 (0.6) | 173 (23.7) | 168 (22.0) | 124 (21.4) | |
| Hb: 11-11.9 g/dL | 313 (15.1) | 64 (12.4) | 112 (20.4) | 122 (22.6) | 15 (3.2) | 134 (18.3) | 109 (14.3) | 70 (12.1) | |
| Hb: 12-12.9 g/dL | 256 (12.3) | 33 (6.4) | 49 (8.9) | 83 (15.4) | 91 (19.4) | 103 (14.1) | 100 (13.1) | 53 (9.1) | |
| Hb ≥13 g/dL | 454 (21.9) | 8 (1.5) | 34 (6.2) | 53 (9.8) | 359 (76.7) | 154 (21.1) | 177 (23.2) | 123 (21.2) | |

DRC, Democratic Republic of Congo; Hb, Hemoglobin; SD, standard deviation; ; WHO, World Health Organization; Arm 1: Consumed nutrition supplement before and during pregnancy; Arm 2: Consumed nutrition supplement during pregnancy; Arm 3: control arm.

| **Supplemental Table 3b: Distribution of hemoglobin at 32 weeks of gestation by Study Site and Intervention Arm** | | | | | | | | | |
| --- | --- | --- | --- | --- | --- | --- | --- | --- | --- |
|  | **All**  **(n=2157)** | **India (n=570)** | **Pakistan (n=614)** | **DRC (n=436)** | **Guatemala (n=537)** | **Arm 1 (n=702)** | **Arm 2 (n=757)** | **Arm 3**  **(n=698)** |  |
| **Hemoglobin (Hb: g/dL), mean ± SD** | 10.8±2.0 | 10.1±1.0 | 9.3±1.7 | 10.7±1.4 | 13.2±1.3 | 10.8±2.0 | 10.8±2.0 | 10.8±2.0 |  |
| **Hemoglobin Status (Cutoff: 11 g/dL), n (%)** |  |  |  |  |  |  |  |  |  |
| Anemia (Hb <11 g/dL) | 1251 (60.3) | 472 (82.8) | 504 (82.1) | 252 (57.8) | 23 (4.3) | 409 (58.3) | 428 (56.5) | 414 (59.3) |  |
| No anemia (Hb ≥11g/dL) | 906 (43.7) | 98 (17.2) | 110 (17.9) | 184 (42.2) | 514 (95.7) | 293 (41.7) | 329 (43.5) | 284 (40.7) |  |
| **Anemia (WHO classification)** (2)**, n (%)** |  |  |  |  |  |  |  |  |  |
| No anemia (Hb ≥11 g/dL) | 906 (43.7) | 98 (17.2) | 110 (17.9) | 184 (42.2) | 514 (95.7) | 293 (41.7) | 329 (43.5) | 284 (40.7) |  |
| Mild Anemia (Hb: 10-10.9 g/dL) | 532 (25.6) | 264 (46.3) | 113 (18.4) | 136 (31.2) | 19 (3.5) | 186 (26.5) | 184 (24.3) | 162 (23.2) |  |
| Moderate anemia (Hb: 7-9.9 g/dL) | 647 (31.2) | 207 (36.3) | 325 (52.9) | 111 (25.5) | 4 (0.7) | 198 (28.2) | 221 (29.2) | 228 (32.7) |  |
| Severe anemia (HB < 7 g/dL) | 72 (3.5) | 1 (0.2) | 66 (10.7) | 5 (1.1) | 0 (0.0) | 25 (3.6) | 23 (3.0) | 24 (3.4) |  |
| **HB cut-offs, n (%)** |  |  |  |  |  |  |  |  |  |
| Hb: <7 g/dL | 72 (3.5) | 1 (0.2) | 66 (10.7) | 5 (1.1) | 0 (0.0) | 25 (3.6) | 23 (3.0) | 24 (3.4) |  |
| Hb: 7-7.9 g/dL | 78 (3.8) | 9 (1.6) | 62 (10.1) | 6 (1.4) | 0 (0.0) | 26 (3.7) | 30 (4.0) | 22 (3.2) |  |
| Hb: 8-8.9 g/dL | 187 (9.0) | 34 (6.0) | 125 (20.4) | 28 (6.4) | 1 (0.2) | 53 (7.5) | 66 (8.7) | 68 (9.7) |  |
| Hb: 9-9.9 g/dL | 382 (18.4) | 164 (28.8) | 138 (22.5) | 77 (17.7) | 3 (0.6) | 119 (17.0) | 125 (16.5) | 138 (19.8) |  |
| Hb: 10-10.9 g/dL | 532 (25.6) | 264 (46.3) | 113 (18.4) | 136 (31.2) | 19 (3.5) | 186 (26.5) | 184 (24.3) | 162 (23.2) |  |
| Hb: 11-11.9 g/dL | 284 (13.7) | 65 (11.4) | 74 (12.1) | 102 (23.4) | 43 (8.0) | 97 (13.8) | 99 (13.1) | 88 (12.6) |  |
| Hb: 12-12.9 g/dL | 250 (12.0) | 29 (5.1) | 25 (4.1) | 56 (12.8) | 140 (26.1) | 77 (11.0) | 102 (13.5) | 71 (10.2) |  |
| Hb ≥13 g/dL | 372 (17.9) | 4 (0.7) | 11 (1.8) | 26 (6.0) | 331 (61.6) | 119 (17.0) | 128 (16.9) | 125 (17.9) |  |

DRC, Democratic Republic of Congo; Hb, Hemoglobin; SD, standard deviation; ; WHO, World Health Organization; Arm 1: Consumed nutrition supplement before and during pregnancy; Arm 2: Consumed nutrition supplement during pregnancy; Arm 3: control arm.


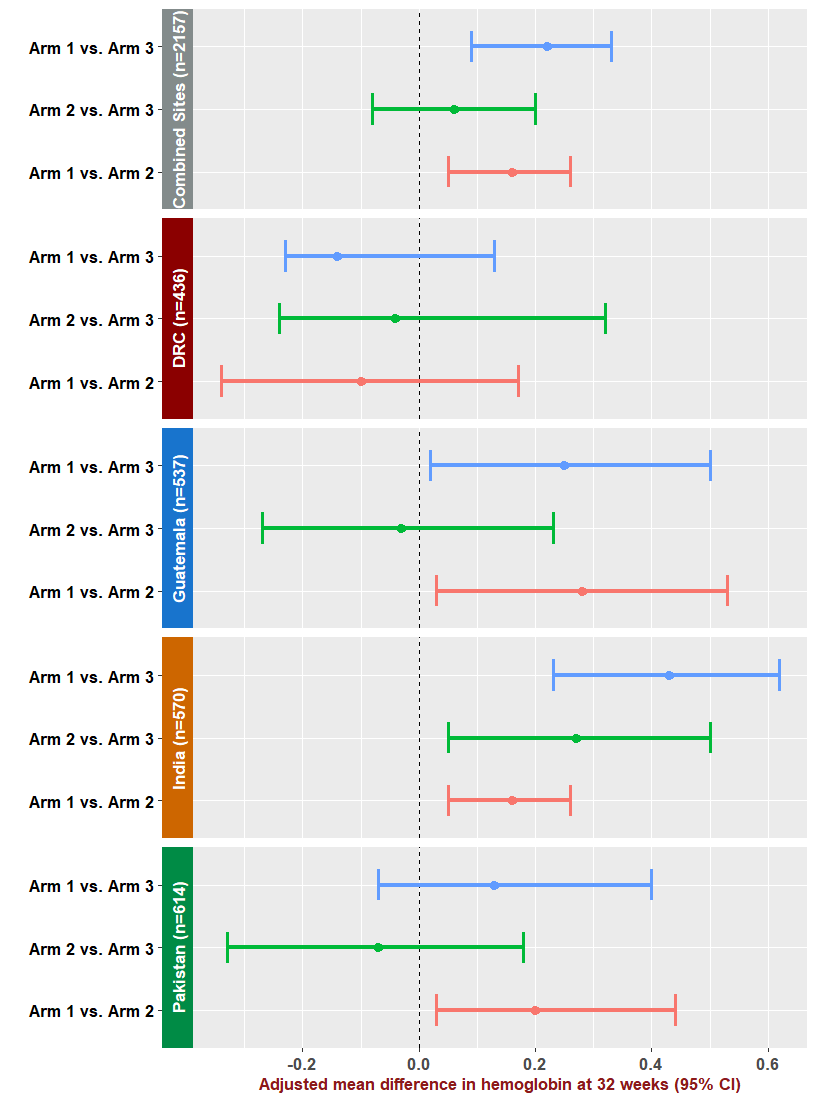


**Supplemental Figure 2:** Effect of small quantity lipid-based nutrient supplement (X) on hemoglobin (M) at 32 weeks of gestation: X-M association for combined and site-specific analysis at 32 weeks of gestation. The graph represents adjusted mean difference (beta-coefficients) in hemoglobin (g/dL) at 32 weeks of gestation between the randomized Arms.

Arm 1: Consumed nutrition supplement before and during pregnancy; Arm 2: Consumed nutrition supplement during pregnancy; Arm 3: control arm. Combined analysis: Linear models were used to compute mean differences in hemoglobin at 32 weeks after adjusting for site, cluster, maternal age, parity, education, socioeconomic status, pre-pregnancy hemoglobin, and pre-pregnancy body mass index. For site-specific analyses: Linear models were used to compute mean differences in hemoglobin at 32 weeks after adjusting for cluster, maternal age, parity, education, socioeconomic status, pre-pregnancy hemoglobin, and pre-pregnancy body mass index.


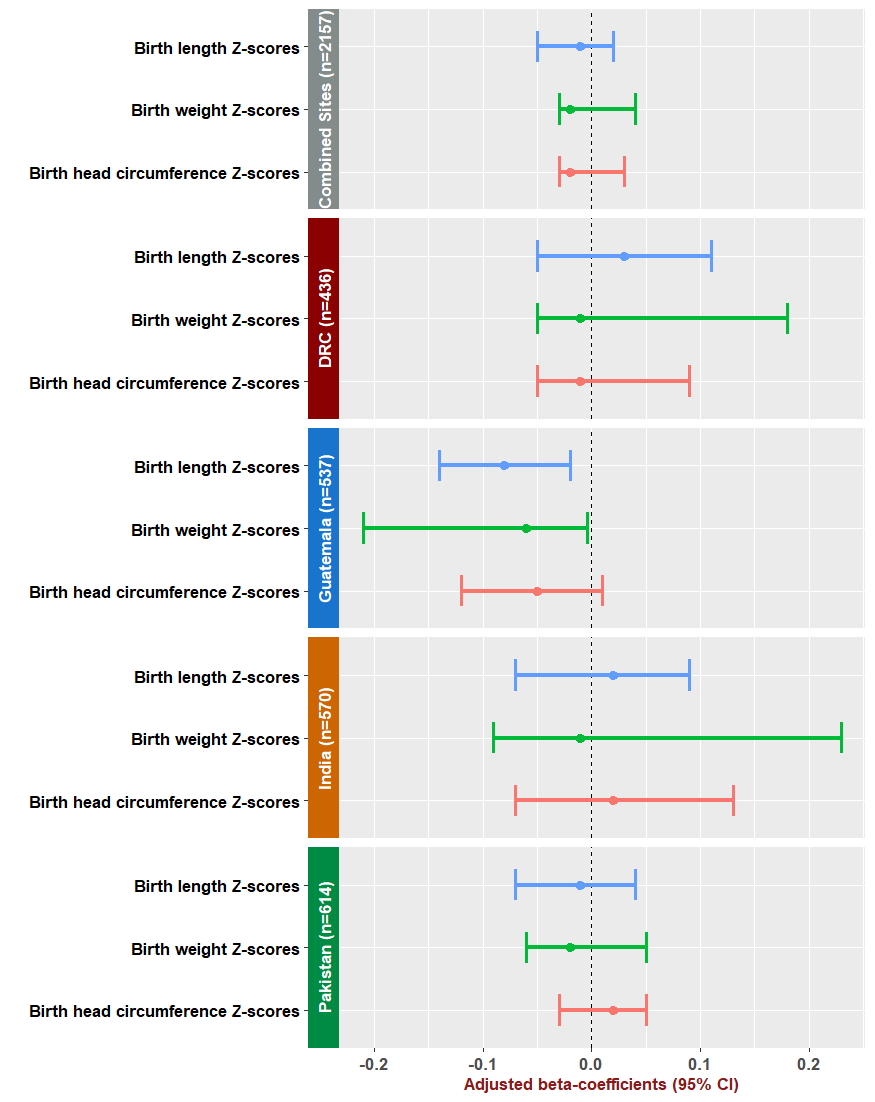


**Supplemental Figure 3**: Relationship between hemoglobin (g/dL) at 32 weeks of gestation (Mediator: M) and three markers of intrauterine growth (Outcome: Y): M-Y association at 32 weeks of gestation. The graph represents adjusted regression co-efficient for change in Z-scores for birth length, birth weight, and birth head circumference -for-age (Y) associated with one gram /dL increase in hemoglobin (M) at 32 weeks of gestation: Combined and site-specific analysis for mediator-outcome (M-Y) association at 32 weeks of gestation. M-Y confounders included in the analysis were cluster, maternal age, parity, education, socioeconomic status, pre-pregnancy hemoglobin, and pre-pregnancy body mass index.

| **Supplemental Table 4: Decomposition of total effect of preconception small quantity lipid-based nutrition supplement on three markers of intrauterine growth into direct and indirect effects- Arm 1 (preconception) vs. Arm 3 (control) comparison at 12 weeks of gestation** | | | | | | | | | | |
| --- | --- | --- | --- | --- | --- | --- | --- | --- | --- | --- |
| **Adjusted mean differences in Z-scores (95% CIs) for birth length-for-age** | | | | | | | | | | |
|  | ***Direct effect*** | | | ***Indirect Effect*** | | | | ***Total Effect*** | | |
|  | **Beta-coefficient** | **lower CI** | **Upper CI** | **Beta-coefficient** | **lower CI** | **Upper CI** | **Beta-coefficient** | | **lower CI** | **Upper CI** |
| **Combined** | 0.18 | 0.09 | 0.33 | 0.02 | -0.02 | 0.01 | 0.20 | | 0.09 | 0.33 |
| **DRC** | 0.32 | 0.07 | 0.56 | 0.001 | -0.02 | 0.01 | 0.32 | | 0.08 | 0.55 |
| **Guatemala** | -0.10 | -0.28 | 0.04 | 0.004 | -0.01 | 0.06 | -0.10 | | -0.26 | 0.06 |
| **India** | 0.21 | -0.01 | 0.46 | 0.02 | -0.07 | 0.06 | 0.23 | | -0.01 | 0.44 |
| **Pakistan** | 0.24 | -0.01 | 0.48 | 0.06 | -0.001 | 0.12 | 0.30 | | 0.04 | 0.53 |
| **Adjusted mean differences in Z-scores (95% CIs) for birth weight-for-age** | | | | | | | | | | |
| **Combined** | 0.12 | 0.03 | 0.23 | 0.01 | -0.01 | 0.02 | 0.13 | | 0.03 | 0.23 |
| **DRC** | 0.21 | 0.05 | 0.41 | 0.0002 | -0.02 | 0.02 | 0.21 | | 0.06 | 0.41 |
| **Guatemala** | -0.06 | -0.21 | 0.13 | 0.01 | -0.03 | 0.03 | -0.05 | | -0.23 | 0.14 |
| **India** | 0.21 | -0.08 | 0.34 | 0.01 | -0.06 | 0.08 | 0.22 | | 0.01 | 0.42 |
| **Pakistan** | 0.17 | -0.01 | 0.33 | 0.02 | -0.0200 | 0.08 | 0.19 | | -0.01 | 0.37 |
| **Adjusted mean differences in Z-scores (95% CIs) for birth head circumference-for-age** | | | | | | | | | | |
| **Combined** | 0.06 | -0.03 | 0.20 | 0.01 | -0.01 | 0.02 | 0.07 | | -0.03 | 0.20 |
| **DRC** | 0.08 | -0.23 | 0.28 | 0.004 | -0.01 | 0.02 | 0.08 | | -0.23 | 0.28 |
| **Guatemala** | -0.09 | -0.31 | 0.12 | 0.004 | -0.03 | 0.04 | -0.09 | | -0.29 | 0.12 |
| **India** | 0.17 | -0.08 | 0.40 | -0.02 | -0.10 | 0.05 | 0.15 | | -0.06 | 0.35 |
| **Pakistan** | 0.03 | -0.14 | 0.21 | 0.06 | 0.01 | 0.12 | 0.09 | | -0.10 | 0.27 |

DRC, Democratic Republic of Congo; WHO, World Health Organization

Arm 1 vs. Arm 3: Comparison of women who consumed small quantity lipid-based nutrition supplement from preconception until birth (Arm 1) vs. women who did not take small quantity lipid-based nutrition supplement at all (Arm 3: control Arm)

Z-scores for birth length, weight and head circumference-for-age are based on WHO child growth standards that account for newborn’s sex and age (non-gestational age adjusted data) (3). Two way-decomposition of total effect into direct and indirect effect (mediated by hemoglobin at 12 weeks of pregnancy) was done after adjusting for mediator outcome (M-Y) confounders: cluster, maternal age, parity, education, socioeconomic status, pre-pregnancy hemoglobin, and pre-pregnancy body mass index.

| **Supplemental Table 5: Decomposition of total effect of preconception lipid-based nutrition supplement on three markers of intrauterine growth into direct and indirect effects: Arm 1 (preconception) vs. Arm 3 (control) comparison at 32 weeks of gestation** | | | | | | | | | | |
| --- | --- | --- | --- | --- | --- | --- | --- | --- | --- | --- |
| **Adjusted mean differences in z-scores (95% CIs) for birth length-for-age** | | | | | | | | | | |
|  | ***Direct effect*** | | | ***Indirect Effect*** | | | | ***Total Effect*** | | |
|  | **Beta-coefficient** | **lower CI** | **Upper CI** | **Beta-coefficient** | **lower CI** | **Upper CI** | **Beta-coefficient** | | **lower CI** | **Upper CI** |
| **Combined** | 0.21 | 0.10 | 0.34 | -0.002 | -0.01 | 0.01 | 0.21 | | 0.09 | 0.33 |
| **DRC** | 0.32 | 0.08 | 0.52 | -0.001 | -0.03 | 0.02 | 0.32 | | 0.08 | 0.52 |
| **Guatemala** | -0.08 | -0.31 | 0.12 | -0.02 | -0.01 | 0.04 | -0.10 | | -0.36 | 0.09 |
| **India** | 0.22 | 0.02 | 0.46 | 0.01 | -0.04 | 0.05 | 0.23 | | 0.03 | 0.46 |
| **Pakistan** | 0.31 | 0.07 | 0.58 | -0.001 | -0.02 | 0.01 | 0.31 | | 0.07 | 0.58 |
| **Adjusted mean differences in z-scores (95% CIs) for birth weight-for-age** | | | | | | | | | | |
| **Combined** | 0.16 | 0.07 | 0.27 | -0.0042 | -0.009 | 0.002 | 0.16 | | 0.07 | 0.27 |
| **DRC** | 0.21 | 0.04 | 0.44 | 0.0005 | -0.02 | 0.02 | 0.21 | | 0.04 | 0.44 |
| **Guatemala** | -0.02 | -0.21 | 0.17 | -0.0162 | -0.05 | 0.0001 | -0.04 | | -0.22 | 0.15 |
| **India** | 0.22 | 0.03 | 0.44 | -0.0046 | -0.04 | 0.05 | 0.22 | | 0.04 | 0.41 |
| **Pakistan** | 0.18 | -0.01 | 0.38 | -0.0026 | -0.01 | 0.01 | 0.18 | | -0.01 | 0.38 |
| **Adjusted mean differences in z-scores (95% CIs) for birth head circumference-for-age** | | | | | | | | | | |
| **Combined** | 0.10 | -0.01 | 0.22 | -0.0042 | -0.009 | 0.001 | 0.1 | | -0.01 | 0.22 |
| **DRC** | 0.07 | -0.14 | 0.32 | -0.001 | -0.03 | 0.01 | 0.07 | | -0.14 | 0.32 |
| **Guatemala** | -0.05 | -0.27 | 0.15 | -0.0135 | -0.05 | 0.01 | -0.07 | | -0.28 | 0.12 |
| **India** | 0.13 | -0.06 | 0.35 | 0.0092 | -0.04 | 0.06 | 0.14 | | -0.05 | 0.36 |
| **Pakistan** | 0.11 | -0.12 | 0.3 | 0.0013 | -0.01 | 0.01 | 0.11 | | -0.12 | 0.31 |

DRC, Democratic Republic of Congo; WHO, World Health Organization

Arm 1 vs. Arm 3: Comparison of women who consumed small quantity lipid-based nutrition supplement from preconception until birth (Arm 1) vs. women who did not take a small quantity lipid-based nutrition supplement at all (Arm 3: control Arm)

Z-scores for birth length, weight and head circumference-for-age are based on the WHO child growth standards that account for newborn’s sex and age (non-gestational age adjusted data) (3).

Two way-decomposition of total effect into direct and indirect effect (mediated by hemoglobin at 32 weeks of pregnancy) was done after adjusting for mediator-outcome (M-Y) confounders: maternal age, parity, education, socioeconomic status, pre-pregnancy hemoglobin, and pre-pregnancy body mass index.

| **Supplemental Table 6: Decomposition of total effect of small quantity lipid-based nutrition supplement on three markers of intrauterine growth into direct and indirect effects: Arm 2 (during pregnancy) vs. Arm 3 (control) comparison at 32 weeks of gestation** | | | | | | | | | | |
| --- | --- | --- | --- | --- | --- | --- | --- | --- | --- | --- |
| **Adjusted mean differences in Z-scores (95% CIs) for birth length-for-age** | | | | | | | | | | |
|  | **Direct effect** | | | **Indirect Effect** | | | | **Total Effect** | | |
|  | **Beta-coefficient** | **lower CI** | **Upper CI** | **Beta-coefficient** | **lower CI** | **Upper CI** | **Beta-coefficient** | | **lower CI** | **Upper CI** |
| **Combined** | 0.22 | 0.11 | 0.33 | -0.0006 | -0.009 | 0.005 | 0.22 | | 0.11 | 0.33 |
| **DRC** | 0.19 | -0.04 | 0.4 | 0.0012 | -0.02 | 0.02 | 0.19 | | -0.03 | 0.4 |
| **Guatemala** | 0.19 | -0.01 | 0.37 | 0.0016 | -0.03 | 0.02 | 0.19 | | -0.02 | 0.37 |
| **India** | 0.22 | -0.01 | 0.42 | 0.0058 | -0.02 | 0.04 | 0.23 | | 0.01 | 0.43 |
| **Pakistan** | 0.21 | -0.03 | 0.45 | 0.0009 | -0.01 | 0.02 | 0.21 | | -0.03 | 0.45 |
| **Adjusted mean differences in Z-scores (95% CIs) for birth weight-for-age** | | | | | | | | | | |
| **Combined** | 0.15 | 0.06 | 0.24 | -0.001 | -0.004 | 0.003 | 0.15 | | 0.06 | 0.24 |
| **DRC** | 0.13 | -0.06 | 0.31 | 0.000 | -0.01 | 0.01 | 0.13 | | -0.06 | 0.31 |
| **Guatemala** | 0.14 | -0.02 | 0.29 | 0.001 | -0.02 | 0.02 | 0.14 | | -0.02 | 0.29 |
| **India** | 0.18 | -0.01 | 0.38 | -0.003 | -0.03 | 0.02 | 0.18 | | -0.01 | 0.38 |
| **Pakistan** | 0.08 | -0.12 | 0.27 | 0.002 | -0.01 | 0.01 | 0.08 | | -0.11 | 0.27 |
| **Adjusted mean differences in Z-scores (95% CIs) for birth head circumference-for-age** | | | | | | | | | | |
| **Combined** | 0.09 | -0.02 | 0.21 | -0.001 | -0.004 | 0.004 | 0.09 | | -0.02 | 0.22 |
| **DRC** | -0.03 | -0.24 | 0.17 | 0.001 | -0.02 | 0.02 | -0.03 | | -0.24 | 0.17 |
| **Guatemala** | 0.11 | -0.07 | 0.33 | 0.001 | -0.02 | 0.01 | 0.11 | | -0.07 | 0.32 |
| **India** | 0.10 | -0.09 | 0.34 | 0.006 | -0.02 | 0.03 | 0.11 | | -0.1 | 0.35 |
| **Pakistan** | 0.10 | -0.12 | 0.33 | -0.001 | -0.01 | 0.01 | 0.1 | | -0.12 | 0.33 |

Abbreviations: DRC, Democratic Republic of Congo; WHO, World Health Organization

Arm 2 vs. Arm 3: Comparison of women who consumed a small quantity lipid-based nutrition supplement from the second trimester of pregnancy until birth (Arm 2) vs. women who did not take the supplement (Arm 3: control Arm)

Z-scores for birth length, weight, and head circumference -for-age are based on the WHO child growth standards that account for newborn’s sex and age (non-gestational age adjusted data).

Two way-decomposition of total effect into direct and indirect effect (mediated by hemoglobin at 32 weeks of pregnancy) was done after adjusting for mediator-outcome (M-Y) confounders: maternal age, parity, education, socioeconomic status, pre-pregnancy hemoglobin, and pre-pregnancy body mass index.

**Supplemental Table 7.** **Decomposition of total effect of preconception small quantity lipid-based nutrition supplement on three markers of intrauterine growth into direct and indirect effects: Findings after adjusting for gestational age (WHO child growth vs. INTERGROWTH fetal growth standards) (3, 4)**

| **12 weeks of gestation (Arm 1 vs. Arm 3)** | | | |
| --- | --- | --- | --- |
| **WHO growth standards (non-gestational age adjusted data)** | | | |
|  | **Direct effect** | **Indirect effect** | **Total effect** |
| **Birth length Z-scores** | 0.18 (0.09, 0.33) | 0.02 (-0.02, 0.01) | 0.20 (0.09, 0.33) |
| **Birth weight Z-scores** | 0.12 (0.03, 0.23) | 0.01 (-0.01, 0.02) | 0.13 (0.03, 0.23) |
| **Birth head circumference Z-scores** | 0.06 (-0.03, 0.20) | 0.01 (-0.01, 0.02) | 0.07 (-0.03, 0.20) |
| **INTERGROWTH standards (gestational age adjusted data)** | | | |
| **Birth length Z-scores** | 0.23 (0.09, 0.36) | 0.002 (-0.04, 0.04) | 0.23 (0.10, 0.36) |
| **Birth weight Z-scores** | 0.21 (0.09, 0.34) | 0.01 (-0.02, 0.05) | 0.22 (0.11, 0.34) |
| **Birth head circumference Z-scores** | 0.07 (-0.07, 0.21) | 0.01 (-0.03, 0.05) | 0.08 (-0.05, 0.22) |
| **32 weeks of gestation (Arm 1 vs. Arm 3)** | | | |
| **WHO growth standards (non-gestational age adjusted data)** | | | |
| **Birth length Z-scores** | 0.21 (0.10, 0.34) | -0.002 (-0.01, 0.01) | 0.21 (0.09, 0.33) |
| **Birth weight Z-scores** | 0.16 (0.07, 0.27) | -0.004 (-0.01, 0.002) | 0.16 (0.07, 0.27) |
| **Birth head circumference Z-scores** | 0.10 (-0.01, 0.22) | -0.004 (-0.01, 0.001) | 0.10 (-0.01. 0.22) |
| **INTERGROWTH standards (gestational age adjusted data)** | | | |
| **Birth length Z-scores** | 0.24 (0.12, 0.37) | -0.01 (-0.03, 0.003) | 0.23 (0.10, 0.36) |
| **Birth weight Z-scores** | 0.23 (0.12, 0.35) | -0.01 (-0.02, 0.004) | 0.22 (0.11, 0.34) |
| **Birth head circumference Z-scores** | 0.08 (-0.04, 0.22) | -0.009 (-0.02, 0.005) | 0.07 (-0.05, 0.22) |

DRC, Democratic Republic of Congo; WHO, World Health Organization

Arm 1 vs. Arm 3: Comparison of women who consumed a small quantity lipid-based nutrition supplement from preconception until birth (Arm 1) vs. women who did not take the supplement at all (Arm 3: control Arm)

Decomposition of the total effect into natural direct and indirect effect was done after adjusting for mediator-outcome (M-Y) confounders: Site, maternal age, parity, education, socioeconomic status, pre-pregnancy hemoglobin, and pre-pregnancy body mass index.

The data on gestational age were available from three countries including Pakistan, India, and Guatemala. Gestational age was not measured in the DRC due to lack of trained personnel.

| ***Simple Models (No interaction terms)^1^*** | | | | | | | | | | | | | | | | | | |
| --- | --- | --- | --- | --- | --- | --- | --- | --- | --- | --- | --- | --- | --- | --- | --- | --- | --- | --- |
|  | | **Direct effect** | | | | | | **Indirect effect** | | | | | | **Total effect** | | | | |
|  | | **Beta-coefficient** | | **lower CI** | | **Upper CI** | | **Beta-coefficient** | | **lower CI** | | **Upper CI** | | **Beta-coefficient** | | **lower CI** | | **Upper CI** |
| **Birth length Z-scores** | 0.18 | | 0.09 | | 0.33 | | 0.02 | | -0.02 | | 0.01 | | 0.20 | | 0.09 | | 0.33 | |
| **Birth weight Z-scores** | 0.12 | | 0.03 | | 0.23 | | 0.01 | | -0.01 | | 0.02 | | 0.13 | | 0.03 | | 0.23 | |
| **Birth head circumference Z-scores** | 0.06 | | -0.03 | | 0.2 | | 0.01 | | -0.01 | | 0.02 | | 0.07 | | -0.03 | | 0.2 | |
| ***Complex model 1: Interaction between intervention Arm and Hb at 12 weeks of gestation (Arm*Hb_12)***^2^ | | | | | | | | | | | | | | | | | | |
| **Birth length Z-scores** | | 0.22 | | 0.09 | | 0.35 | | -0.02 | | -0.05 | | 0.01 | | 0.20 | | 0.08 | | 0.32 |
| **Birth weight Z-scores** | | 0.14 | | 0.04 | | 0.25 | | -0.01 | | -0.04 | | 0.10 | | 0.13 | | 0.02 | | 0.23 |
| **Birth head circumference Z-scores** | | 0.08 | | -0.05 | | 0.19 | | -0.01 | | -0.04 | | 0.01 | | 0.07 | | -0.07 | | 0.17 |
| ***Complex model 2: Interaction between Arm and Hb at baseline (Arm*Hb_baseline)***^2^ | | | | | | | | | | | | | | | | | | |
| **Birth length Z-scores** | | 0.2 | | 0.1 | | 0.32 | | -0.001 | | -0.01 | | 0.02 | | 0.2 | | 0.09 | | 0.32 |
| **Birth weight Z-scores** | | 0.13 | | 0.03 | | 0.25 | | 0.001 | | -0.01 | | 0.02 | | 0.13 | | 0.02 | | 0.24 |
| **Birth head circumference Z-scores** | | 0.07 | | -0.07 | | 0.17 | | 0.001 | | -0.01 | | 0.02 | | 0.07 | | -0.07 | | 0.17 |
| ***Complex model 3: (Arm*Hb_12) and (Arm*Hb_baseline)***^2^ | | | | | | | | | | | | | | | | | | |
| **Birth length Z-scores** | | 0.22 | | 0.07 | | 0.34 | | -0.01 | | -0.05 | | 0.01 | | 0.21 | | 0.08 | | 0.34 |
| **Birth weight Z-scores** | | 0.13 | | 0.05 | | 0.25 | | -0.003 | | -0.03 | | 0.02 | | 0.13 | | 0.05 | | 0.23 |
| **Birth head circumference Z-scores** | | 0.07 | | -0.04 | | 0.22 | | -0.003 | | -0.03 | | 0.03 | | 0.07 | | -0.05 | | 0.21 |

**Supplemental Table 8: Direct, indirect, and total/main effect of small quantity lipid-based nutrient supplement on the three markers of intrauterine growth: Simple versus complex models for Arm 1 vs. Arm 3 at 12 weeks of gestation**

Hb, Hemoglobin; WHO, World Health Organization

Arm 1 vs. Arm 3: Comparison of women who consumed a small quantity lipid-based nutrition supplement from preconception until birth (Arm 1) vs. women who did not take the supplement at all (Arm 3: control Arm).

Z-scores for birth length, weight, and head circumference-for-age are based on WHO child growth standards that account for newborn’s sex and age (non-gestational age adjusted data) (3).

^1^Simple model was multivariable model that included no interaction terms in the model. The model included mediator-outcome (M-Y) confounders including site, maternal age, parity, education maternal baseline BMI, and baseline hemoglobin (Hb).

^2^Complex models included mediator-outcome M-Y confounders (site, maternal age, parity, education maternal baseline BMI, and baseline Hb) and interaction terms between Arm and Hb at 12 weeks (complex model 1), interaction between Arm and Hb at baseline (complex model 2), and both interactions terms simultaneously (complex model 3).

| **Supplemental Table 9: Four-way decomposition of total effect of small quantity lipid-based nutrition supplement on three markers of intrauterine growth: Complex models with linear and quadratic forms of hemoglobin and interaction terms- All sites combined** | | | | | |
| --- | --- | --- | --- | --- | --- |
| **Markers of intrauterine growth** | **Adjusted mean differences in Z-scores and 95% CIs: *Arm 1 vs. Arm 3*** | | | | |
|  | ***At 12 weeks of gestation (n=2075)*** | | | | |
|  | ***Natural direct effect (NDE)*** | | ***Natural indirect effect (NIE)*** | | ***Total/main effect*** |
|  | ***CDE*** | ***Int_ref_*** | ***PIE*** | ***Int_Med_*** |  |
| **Birth length Z-scores** | 0.20 (0.08, 0.31) | 0.02 (-0.003, 0.04) | 0.02 (-0.01, 0.04) | -0.04 (-0.07, 0.005) | 0.20 (0.07, 0.32) |
| **Birth weight Z-scores** | 0.13 (0.03, 0.25) | 0.01 (-0.005, 0.03) | 0.01 (-0.01, 0.04) | -0.02 (-0.05, 0.01) | 0.13 (0.01, 0.24) |
| **Birth head circumference Z-scores** | 0.07 (-0.05, 0.18) | 0.01 (-0.006, 0.04) | 0.01 (-0.01, 0.03) | -0.02 (-0.06, 0.01) | 0.07 (-0.05, 0.18) |
|  | ***At 32 weeks of gestation (n=2157)*** | | | | |
| **Birth length Z-scores** | 0.21 (0.07, 0.32) | 0.004 (-0.005, 0.02) | 0.001 (-0.005, 0.01) | -0.01 (-0.04, -0.001) | 0.20 (0.08, 0.33) |
| **Birth weight Z-scores** | 0.15 (0.06, 0.24) | 0.002 (-0.007, 0.01) | 0.001 (-0.01, 0.01) | -0.01 (-0.03, 0.001) | 0.14 (0.05, 0.23) |
| **Birth head circumference Z-scores** | 0.07 (-0.04, 0.17) | 0.003 (-0.004, 0.01) | -0.0005 (-0.01, 0.01) | -0.01 (-0.02, 0.001) | 0.07 (-0.05, 0.16) |
|  | **Adjusted mean differences in Z-scores and 95% CIs: *Arm 2 vs. Arm 3 at 32 weeks of gestation (n=2157)*** | | | | |
| **Birth length Z-scores** | 0.21 (0.11, 0.30) | 0.001 (-0.004, 0.01) | 0.0001 (-0.003, 0.004) | -0.0003 (-0.006, 0.003) | 0.21 (0.11, 0.31) |
| **Birth weight Z-scores** | 0.14 (0.03, 0.23) | -0.0002 (-0.006, 0.004) | 0.0001 (-0.003, 0.003) | 0.0001 (-0.006, 0.004) | 0.14 (0.05, 0.22) |
| **Birth head circumference Z-scores** | 0.09 (-0.02, 0.20) | -0.0005 (-0.004, 0.006) | -0.0001 (-0.004,0.003) | 0.0001 (-0.004,0.006) | 0.09 (-0.02, 0.16) |

CDE; controlled direct effect, Hb, Hemoglobin; IntMed; Mediated interaction; Intref, reference interaction; NDE, Natural direct effect; NIE, Natural indirect effect PIE, Pure indirect effect; WHO, World Health Organization; 95% CIs, 95% confidence intervals

CDE; controlled direct effect: component of total effect neither due to mediation nor interaction; Intref; reference interaction: component just due to interaction; PIE; pure indirect effect: a pure mediated effect; IntMed; Mediated interaction: component due to both mediation and interaction.

CDE and Intref are added to compute the natural direct effect (NDE); PIE and IntMed are added to generate the natural indirect effect (NIE)

Arm 1 vs. Arm 3: Comparison of women who consumed a small quantity lipid-based nutrient supplement from preconception until birth (Arm 1) vs. women who did not take the supplement at all (Arm 3: control Arm)

Arm 2 vs. Arm 3: Comparison of women who consumed nutrient supplements from the second trimester of pregnancy until birth (Arm 2) vs. women who did not take the nutrient supplement (Arm 3: control Arm)

Z-scores for birth length, weight, and head circumference -for-age are based on WHO child growth standards that account for newborn’s sex and age (non-gestational age adjusted data) (3).

Decomposition of the total effect into natural direct and indirect effect was done after keeping interaction terms (Arm* Hb during pregnancy and Arm*Pre-pregnancy Hb) and adjusting for mediator-outcome (M-Y) confounders: Site, Cluster, maternal age, parity, education, socioeconomic status, pre-pregnancy hemoglobin, and pre-pregnancy body mass index. We included both linear and quadratic terms of hemoglobin (both were centered to mean) and their interaction terms with intervention Arm.

| **Supplemental Table 10: Four-way decomposition of total effect of small quantity lipid-based nutrition supplement on three markers of intrauterine growth: Complex models with linear and quadratic forms of Hb and interaction terms- Pakistan** | | | | | | | | | |
| --- | --- | --- | --- | --- | --- | --- | --- | --- | --- |
| **Markers of intrauterine growth** | **Adjusted mean differences in z-scores and 95% CIs: *Arm 1 vs. Arm 3*** | | | | | | | | |
|  | ***At 12 weeks of gestation (n=550)*** | | | | | | | | |
|  | ***Natural direct effect (NDE)*** | | | ***Natural indirect effect (NIE)*** | | | | ***Total/main effect*** | |
|  | ***CDE*** | ***Int_ref_*** | | ***PIE*** | | ***Int_Med_*** | |  |  |
| **Birth length Z-scores** | 0.22 (-0.03, 0.49) | | 0.02 (-0.07, 0.12) | | 0.08 (-0.05, 0.22) | | -0.03 (-0.20, 0.12) | | 0.29 (0.02, 0.53) |
| **Birth weight Z-scores** | 0.17 (-0.001, 0.36) | | 0.01 (-0.05, 0.08) | | 0.02 (-0.05, 0.11) | | -0.02 (-0.15, 0.11) | | 0.20 (0.03, 0.40) |
| **Birth head circumference Z-scores** | 0.05 (-0.19, 0.22) | | 0.02 (-0.02, 0.10) | | 0.06 (0.002, 0.23) | | -0.03 (-0.18, 0.03) | | 0.09 (-0.12, 0.30) |
|  | ***At 32 weeks of gestation (n=614)*** | | | | | | | | |
| **Birth length Z-scores** | 0.30 (0.10, 0.55) | -0.002 (-0.02, 0.02) | | 0.007(-0.01, 0.03) | | -0.01 (-0.05, 0.02) | | 0.29 (0.07, 0.56) | |
| **Birth weight Z-scores** | 0.19 (-0.01, 0.36) | 0.002 (-0.02, 0.34) | | 0.001 (-0.01, 0.03) | | -0.01 (-0.06, 0.01) | | 0.18 (-0.01, 0.35) | |
| **Birth head circumference Z-scores** | 0.11 (-0.12, 0.27) | 0.0003 (-0.02, 0.03) | | 0.002(-0.01,0.04) | | -0.01 (-0.06, 0.01) | | 0.10 (-0.11, 0.26) | |
|  | **Adjusted mean differences in z-scores and 95% CIs: *Arm 2 vs. Arm 3 at 32 weeks of gestation (n=614)*** | | | | | | | | |
| **Birth length Z-scores** | 0.18(-0.02, 0.40) | -0.002 (-0.03, 0.02) | | -0.005(-0.03, 0.02) | | 0.01 (-0.02, 0.04) | | 0.19 (-0.03, 0.38) | |
| **Birth weight Z-scores** | 0.07 (-0.12, 0.30) | 0.0005 (-0.01, 0.02) | | -0.001 (-0.01, 0.02) | | -0.002 (-0.03, 0.02) | | 0.07 (-0.11, 0.30) | |
| **Birth head circumference Z-scores** | 0.05 (-0.17, 0.25) | 0.001 (-0.02, 0.02) | | -0.001(-0.03,0.02) | | -0.002 (-0.04, 0.02) | | 0.05 (-0.17, 0.24) | |

CDE; controlled direct effect, Hb, Hemoglobin; IntMed; Mediated interaction; Intref, reference interaction; NDE, Natural direct effect; NIE, Natural indirect effect PIE, Pure indirect effect; WHO, World Health Organization; 95% CIs, 95% confidence intervals

CDE; controlled direct effect: component of total effect neither due to mediation nor interaction; Intref; reference interaction: component just due to interaction; PIE; pure indirect effect: a pure mediated effect; IntMed; Mediated interaction: component due to both mediation and interaction.

CDE and Intref are added to compute the natural direct effect (NDE); PIE and IntMed are added to generate the natural indirect effect (NIE)

Arm 1 vs. Arm 3: Comparison of women who consumed a small quantity lipid-based nutrient supplement from preconception until birth (Arm 1) vs. women who did not take the supplement at all (Arm 3: control Arm)

Arm 2 vs. Arm 3: Comparison of women who consumed a small quantity lipid-based nutrient supplement from the second trimester of pregnancy until birth (Arm 2) vs. women who did not take nutrition supplements (Arm 3: control Arm)

Z-scores for birth length, weight, and head circumference -for-age are based on WHO child growth standards that account for newborn’s sex and age (non-gestational age adjusted data) (3).

Decomposition of the total effect into natural direct and indirect effect was done after keeping interaction terms (Arm* Hb during pregnancy and Arm*Pre-pregnancy Hb) and adjusting for mediator-outcome (M-Y) confounders: Cluster, maternal age, parity, education, socioeconomic status, pre-pregnancy hemoglobin, and pre-pregnancy body mass index. We included both linear and quadratic terms of hemoglobin (both were centered to mean) and their interaction terms with intervention Arm.

| **Supplemental Table 11: Four-way decomposition of total effect of small quantity lipid-based nutrition supplement on three markers of intrauterine growth: Complex models with linear and quadratic forms of Hb and interaction terms- India** | | | | | | | | | |
| --- | --- | --- | --- | --- | --- | --- | --- | --- | --- |
| **Markers of intrauterine growth** | **Adjusted mean differences in z-scores and 95% CIs: *Arm 1 vs. Arm 3*** | | | | | | | | |
|  | ***At 12 weeks of gestation (n=518)*** | | | | | | | | |
|  | ***Natural direct effect (NDE)*** | | | ***Natural indirect effect (NIE)*** | | | | ***Total/main effect*** | |
|  | ***CDE*** | ***Int_ref_*** | | ***PIE*** | | ***Int_Med_*** | |  |  |
| **Birth length Z-scores** | 0.21(-0.03, 0.44) | | 0.03 (-0.04, 0.10) | | 0.06 (-0.06, 0.18) | | -0.06 (-0.18, 0.10) | | 0.24 (0.03, 0.45) |
| **Birth weight Z-scores** | 0.18 (-0.04, 0.40) | | 0.03 (-0.03, 0.08) | | 0.08 (-0.06, 0.16) | | -0.07 (-0.17, 0.07) | | 0.22 (0.01, 0.40) |
| **Birth head circumference Z-scores** | 0.16 (-0.10, 0.38) | | -0.01 (-0.05, 0.08) | | -0.02 (-0.13, 0.15) | | 0.01 (-0.18, 0.12) | | 0.15 (-0.10, 0.40) |
|  | ***At 32 weeks of gestation (n=570)*** | | | | | | | | |
| **Birth length Z-scores** | 0.24 (0.10, 0.55) | 0.02 (-0.02, 0.08) | | 0.03(-0.04, 0.11) | | -0.05 (-0.17, 0.04) | | 0.24 (0.02, 043) | |
| **Birth weight Z-scores** | 0.22 (0.02, 0.41) | 0.02 (-0.01, 0.09) | | 0.03 (-0.03, 0.10) | | -0.06 (-0.18, 0.05) | | 0.21 (0.02, 0.41) | |
| **Birth head circumference Z-scores** | 0.14 (-0.10, 0.36) | 0.06 (0.01, 0.10) | | 0.08(0.0005,0.16) | | -0.12 (-0.23, -0.02) | | 0.16 (-0.07, 0.38) | |
|  | **Adjusted mean differences in z-scores and 95% CIs: *Arm 2 vs. Arm 3 at 32 weeks of gestation (n=570)*** | | | | | | | | |
| **Birth length Z-scores** | 0.21(-0.02, 0.40) | 0.04 (-0.02, 0.12) | | 0.03(-0.03, 0.10) | | -0.05 (-0.15, 0.02) | | 0.23 (0.03, 0.41) | |
| **Birth weight Z-scores** | 0.15 (-0.05, 0.34) | 0.04 (-0.02, 0.11) | | 0.03(-0.03, 0.10) | | -0.05 (-0.14, 0.03) | | 0.18 (0.01, 0.37) | |
| **Birth head circumference Z-scores** | 0.10 (-0.11, 0.33) | 0.04 (-0.005, 0.10) | | 0.05(-0.001,0.10) | | -0.05 (-0.14, 0.01) | | 0.11 (-0.13, 0.33) | |

CDE; controlled direct effect, Hb, Hemoglobin; IntMed; Mediated interaction; Intref, reference interaction; NDE, Natural direct effect; NIE, Natural indirect effect PIE, Pure indirect effect; WHO, World Health Organization; 95% CIs, 95% confidence intervals

CDE; controlled direct effect: component of total effect neither due to mediation nor interaction; Intref; reference interaction: component just due to interaction; PIE; pure indirect effect: a pure mediated effect; IntMed; Mediated interaction: component due to both mediation and interaction.

CDE and Intref are added to compute the natural direct effect (NDE); PIE and IntMed are added to generate the natural indirect effect (NIE)

Arm 1 vs. Arm 3: Comparison of women who consumed a small quantity lipid-based nutrient supplement from preconception until birth (Arm 1) vs. women who did not take the supplement at all (Arm 3: control Arm)

Arm 2 vs. Arm 3: Comparison of women who consumed a small quantity lipid-based nutrient supplement from the second trimester of pregnancy until birth (Arm 2) vs. women who did not take nutrition supplements (Arm 3: control Arm)

Z-scores for birth length, birth , and head circumference -for-age are based on WHO child growth standards that account for newborn’s sex and age (non-gestational age adjusted data) (3).

Decomposition of the total effect into natural direct and indirect effect was done after keeping interaction terms (Arm* Hb during pregnancy and Arm*Pre-pregnancy Hb) and adjusting for mediator-outcome (M-Y) confounders: Cluster, maternal age, parity, education, socioeconomic status, pre-pregnancy hemoglobin, and pre-pregnancy body mass index. We included both linear and quadratic terms of Hb (both were centered to mean) and their interaction terms with intervention Arm.

| **Supplemental Table 12: Four-way decomposition of total effect of small quantity lipid-based nutrition supplement on three markers of intrauterine growth: Complex models with linear and quadratic forms of Hb and interaction terms- Democratic Republic of Congo** | | | | | | |
| --- | --- | --- | --- | --- | --- | --- |
| **Markers of intrauterine growth** | **Adjusted mean differences in z-scores and 95% CIs: *Arm 1 vs. Arm 3*** | | | | | |
|  | ***At 12 weeks of gestation (n=539)*** | | | | | |
|  | ***Natural direct effect (NDE)*** | | ***Natural indirect effect (NIE)*** | | ***Total/main effect*** | |
|  | ***CDE*** | ***Int_ref_*** | ***PIE*** | ***Int_Med_*** |  | |
| **Birth length Z-scores** | 0.31 (0.06, 0.50) | -0.001 (-0.04, 0.02) | 0.001 (-0.06, 0.18) | 0.01 (-0.03, 0.07) | | 0.32 (0.08, 0.50) |
| **Birth weight Z-scores** | 0.20 (0.04, 0.42) | -0.004 (-0.03, 0.02) | 0.002 (-0.02, 0.02) | 0.01 (-0.02, 0.06) | | 0.21 (0.04, 0.43) |
| **Birth head circumference Z-scores** | 0.07 (-0.16, 0.31) | -0.01 (-0.04, 0.02) | 0.002 (-0.02, 0.05) | 0.01 (-0.04, 0.06) | | 0.08 (-0.13, 0.32) |
|  | ***At 32 weeks of gestation (n=436)*** | | | | | |
| **Birth length Z-scores** | 0.33 (0.06, 0.54) | -0.01 (-0.04, 0.04) | -0.01(-0.05, 0.03) | 0.01 (-0.04, 0.06) | 0.32 (0.08, 0.50) | |
| **Birth weight Z-scores** | 0.21 (0.02, 0.42) | -0.001 (-0.02, 0.02) | 0.002 (-0.02, 0.02) | 0.001 (-0.04, 0.04) | 0.21 (0.01, 0.44) | |
| **Birth head circumference Z-scores** | 0.07 (-0.16, 0.30) | 0.003 (-0.02, 0.02) | 0.002(-0.03,0.03) | -0.007 (-0.03, 0.03) | 0.07 (-0.15, 0.30) | |
|  | **Adjusted mean differences in z-scores and 95% CIs: *Arm 2 vs. Arm 3 at 32 weeks of gestation (n=436)*** | | | | | |
| **Birth length Z-scores** | 0.19(-0.03, 0.40) | -0.01 (-0.02, 0.02) | -0.001(-0.03, 0.04) | -0.001 (-0.04, 0.02) | 0.18 (-0.03, 0.40) | |
| **Birth weight Z-scores** | 0.13 (-0.08, 0.28) | -0.004 (-0.02, 0.02) | 0.001(-0.01, 0.02) | -0.001 (-0.03, 0.02) | 0.13 (-0.08, 0.28) | |
| **Birth head circumference Z-scores** | -0.03 (-0.26, 0.19) | 0.002 (-0.01, 0.04) | 0.001(-0.02, 0.02) | -0.002 (-0.03, 0.02) | -0.03 (-0.25, 0.18) | |

CDE; controlled direct effect, Hb, Hemoglobin; IntMed; Mediated interaction; Intref, reference interaction; NDE, Natural direct effect; NIE, Natural indirect effect PIE, Pure indirect effect; WHO, World Health Organization; 95% CIs, 95% confidence intervals

CDE; controlled direct effect: component of total effect neither due to mediation nor interaction; Intref; reference interaction: component just due to interaction; PIE; pure indirect effect: a pure mediated effect; IntMed; Mediated interaction: component due to both mediation and interaction.

CDE and Intref are added to compute the natural direct effect (NDE); PIE and IntMed are added to generate the natural indirect effect (NIE)

Arm 1 vs. Arm 3: Comparison of women who consumed a small quantity lipid-based nutrient supplement from preconception until birth (Arm 1) vs. women who did not take the supplement at all (Arm 3: control Arm)

Arm 2 vs. Arm 3: Comparison of women who consumed a small quantity lipid-based nutrient supplement from the second trimester of pregnancy until birth (Arm 2) vs. women who did not take nutrition supplements (Arm 3: control Arm)

Z-scores for birth length, weight, and head circumference -for-age are based on WHO child growth standards that account for newborn’s sex and age (non-gestational age adjusted data) (3).

Decomposition of the total effect into natural direct and indirect effect was done after keeping interaction terms (Arm* Hb during pregnancy and Arm*Pre-pregnancy Hb) and adjusting for mediator-outcome (M-Y) confounders: Cluster, maternal age, parity, education, socioeconomic status, pre-pregnancy hemoglobin, and pre-pregnancy body mass index. We included both linear and quadratic terms of hemoglobin (both were centered to mean) and their interaction terms with intervention Arm.

| **Supplemental Table 13: Four-way decomposition of total effect of small quantity lipid-based nutrition supplement on three markers of intrauterine growth: Complex models with linear and quadratic forms of Hb and interaction terms- Guatemala** | | | | | | | | | |
| --- | --- | --- | --- | --- | --- | --- | --- | --- | --- |
| **Markers of intrauterine growth** | **Adjusted mean differences in z-scores and 95% CIs: *Arm 1 vs. Arm 3*** | | | | | | | | |
|  | ***At 12 weeks of gestation (n=468)*** | | | | | | | | |
|  | ***Natural direct effect (NDE)*** | | | ***Natural indirect effect (NIE)*** | | | | | ***Total/main effect*** |
|  | ***CDE*** | | ***Int_ref_*** | ***PIE*** | | ***Int_Med_*** | | |  |
| **Birth length Z-scores** | -0.10 (-0.31, 0.08) | 0.0001 (-0.03, 0.03) | | | 0.007 (-0.02, 0.06) | | 0.003 (-0.06, 0.06) | -0.10 (-0.29, 0.10) | |
| **Birth weight Z-scores** | -0.05 (-0.21, 0.13) | 0.007 (-0.02, 0.04) | | | 0.01 (-0.03, 0.04) | | -0.02 (-0.07, 0.04) | -0.05 (-0.19, 0.13) | |
| **Birth head circumference Z-scores** | -0.09 (-0.29, 0.10) | 0.01 (-0.02, 0.03) | | | 0.01 (-0.03, 0.05) | | -0.02 (-0.08, 0.04) | -0.09 (-0.26, 0.08) | |
|  | ***At 32 weeks of gestation (n=537)*** | | | | | | | | |
| **Birth length Z-scores** | -0.05 (-0.24, 0.14) | | 0.003 (-0.02, 0.03) | -0.03(-0.07, 0.003) | | -0.02 (-0.08, 0.01) | | | -0.10 (-0.29, 0.10) |
| **Birth weight Z-scores** | -0.03 (-0.21, 0.13) | | 0.005 (-0.02, 0.02) | -0.01 (-0.03, 0.01) | | -0.02 (-0.02, 0.01) | | | -0.05 (-0.23, 0.11) |
| **Birth head circumference Z-scores** | -0.07 (-0.27, 0.12) | | -0.001 (-0.02, 0.01) | -0.01 (-0.04, 0.01) | | -0.005 (-0.06, 0.04) | | | -0.09 (-0.30, 0.08) |
|  | **Adjusted mean differences in z-scores and 95% CIs: *Arm 2 vs. Arm 3 at 32 weeks of gestation (n=537)*** | | | | | | | | |
| **Birth length Z-scores** | 0.18 (0.05, 0.36) | | 0.001 (-0.03, 0.03) | 0.001(-0.03, 0.03) | | -0.001 (-0.04, 0.03) | | | 0.18 (0.03, 0.36) |
| **Birth weight Z-scores** | 0.15 (0.02, 0.30) | | 0.002 (-0.01, 0.02) | 0.003(-0.03, 0.03) | | -0.0002 (-0.02, 0.02) | | | 0.16 (0.03, 0.30) |
| **Birth head circumference Z-scores** | 0.09 (-0.06, 0.30) | | -0.0001 (-0.015, 0.02) | 0.002(-0.02,0.02) | | 0.0005 (-0.02, 0.02) | | | 0.10 (-0.06, 0.28) |

CDE; controlled direct effect, Hb, Hemoglobin; IntMed; Mediated interaction; Intref, reference interaction; NDE, Natural direct effect; NIE, Natural indirect effect PIE, Pure indirect effect; WHO, World Health Organization; 95% CIs, 95% confidence intervals

CDE; controlled direct effect: component of total effect neither due to mediation nor interaction; Intref; reference interaction: component just due to interaction; PIE; pure indirect effect: a pure mediated effect; IntMed; Mediated interaction: component due to both mediation and interaction.

CDE and Intref are added to compute the natural direct effect (NDE); PIE and IntMed are added to generate the natural indirect effect (NIE)

Arm 1 vs. Arm 3: Comparison of women who consumed a small quantity lipid-based nutrient supplement from preconception until birth (Arm 1) vs. women who did not take the supplement at all (Arm 3: control Arm)

Arm 2 vs. Arm 3: Comparison of women who consumed a small quantity lipid-based nutrient supplement from the second trimester of pregnancy until birth (Arm 2) vs. women who did not take nutrition supplements (Arm 3: control Arm)

Z-scores for birth length, weight, and head circumference -for-age are based on WHO child growth standards that account for newborn’s sex and age (non-gestational age adjusted data) (3).

Decomposition of the total effect into natural direct and indirect effect was done after keeping interaction terms (Arm* Hb during pregnancy and Arm*Pre-pregnancy Hb) and adjusting for mediator-outcome (M-Y) confounders: Cluster, maternal age, parity, education, socioeconomic status, pre-pregnancy hemoglobin, and pre-pregnancy body mass index. We included both linear and quadratic terms of hemoglobin (both were centered to mean) and their interaction terms with intervention Arm.

| **Supplemental Table 14: Decomposition of total effect of preconception small quantity lipid-based nutrition supplement on three markers of intrauterine growth into direct and indirect effects- Arm 1 vs. Arm 2+3 comparison at 12 weeks of gestation: *Combined and site-specific analysis*** | | | | | | | | | | |
| --- | --- | --- | --- | --- | --- | --- | --- | --- | --- | --- |
| **Adjusted mean differences in z-scores (95% CIs) for birth length** | | | | | | | | | | |
|  | **Direct effect** | | | **Indirect Effect** | | | | **Total Effect** | | |
|  | **Beta-coefficient** | **lower CI** | **Upper CI** | **Beta-coefficient** | **lower CI** | **Upper CI** | **Beta-coefficient** | | **lower CI** | **Upper CI** |
| **Combined** | 0.08 | -0.01 | 0.20 | 0.01 | -0.01 | 0.02 | 0.09 | | -0.01 | 0.19 |
| **DRC** | 0.23 | 0.04 | 0.41 | -0.001 | -0.01 | 0.01 | 0.23 | | 0.04 | 0.4 |
| **Guatemala** | -0.20 | -0.39 | -0.04 | 0.01 | -0.01 | 0.04 | -0.19 | | -0.37 | -0.03 |
| **India** | 0.11 | -0.08 | 0.34 | 0.01 | -0.04 | 0.06 | 0.12 | | -0.06 | 0.32 |
| **Pakistan** | 0.15 | -0.06 | 0.34 | 0.03 | 0.00 | 0.08 | 0.18 | | -0.01 | 0.37 |
| **Adjusted mean differences in z-scores (95% CIs) for birth weight** | | | | | | | | | | |
| **Combined** | 0.05 | -0.02 | 0.15 | 0.01 | -0.01 | 0.01 | 0.06 | | -0.02 | 0.15 |
| **DRC** | 0.15 | -0.03 | 0.33 | 0.0003 | -0.01 | 0.01 | 0.15 | | -0.03 | 0.33 |
| **Guatemala** | -0.14 | -0.25 | 0.05 | 0.01 | -0.01 | 0.02 | -0.13 | | -0.25 | 0.05 |
| **India** | 0.11 | -0.04 | 0.31 | 0.02 | -0.04 | 0.06 | 0.13 | | -0.03 | 0.31 |
| **Pakistan** | 0.09 | -0.1 | 0.28 | 0.0 | -0.02 | 0.04 | 0.10 | | -0.07 | 0.27 |
| **Adjusted mean differences in z-scores (95% CIs) for birth head circumference** | | | | | | | | | | |
| **Combined** | 0.03 | -0.02 | 0.15 | 0.01 | -0.01 | 0.01 | 0.04 | | -0.05 | 0.14 |
| **DRC** | 0.10 | -0.08 | 0.29 | 0.00 | -0.01 | 0.02 | 0.10 | | -0.08 | 0.29 |
| **Guatemala** | -0.15 | -0.30 | 0.03 | 0.01 | -0.02 | 0.03 | -0.14 | | -0.29 | 0.02 |
| **India** | 0.10 | -0.07 | 0.29 | -0.01 | -0.06 | 0.04 | 0.09 | | -0.10 | 0.28 |
| **Pakistan** | 0.04 | -0.17 | 0.23 | 0.04 | 0.01 | 0.07 | 0.08 | | -0.13 | 0.27 |

DRC, Democratic Republic of Congo; WHO, World Health Organization; 95% CIs, 95% confidence intervals

The results are presented after combining Arm 2 and Arm 2 and comparing with Arm 1.

Arm 1 vs. Arm 2+Arm 3: Comparison of women who consumed a small quantity lipid-based nutrient supplement from preconception until birth (Arm 1) vs. women who started the supplement at 12 weeks of pregnancy (Arm 2) and women who did not take the supplement at all (Arm 3: control Arm)

Z-scores for birth length, weight and head circumference -for-age are based on WHO child growth standards that account for newborn’s sex and age (non-gestational age adjusted data)(3).

Two way-decomposition of total effect into direct and indirect effect (mediated by hemoglobin at 12 weeks of pregnancy) was done after adjusting for mediator-outcome (M-Y) confounders: Cluster, maternal age, parity, education, socioeconomic status, pre-pregnancy hemoglobin, and pre-pregnancy body mass index.

| **Supplemental Table 15: Decomposition of total effect of preconception small quantity lipid-based nutrition supplement on three markers of intrauterine growth into direct and indirect effects- Arm 1 + Arm 2 vs. Arm 3 comparison at 32 weeks of gestation: *Combined and site-specific analysis*** | | | | | | | | | | |
| --- | --- | --- | --- | --- | --- | --- | --- | --- | --- | --- |
| **Adjusted mean differences in z-scores (95% CIs) for birth length** | | | | | | | | | | |
|  | **Direct effect** | | | **Indirect Effect** | | | | **Total Effect** | | |
|  | **Beta-coefficient** | **lower CI** | **Upper CI** | **Beta-coefficient** | **lower CI** | **Upper CI** | **Beta-coefficient** | | **lower CI** | **Upper CI** |
| **Combined** | 0.21 | 0.13 | 0.31 | 0.00 | -0.01 | 0.00 | 0.21 | | 0.13 | 0.31 |
| **DRC** | 0.25 | 0.03 | 0.47 | 0.00 | -0.01 | 0.01 | 0.25 | | 0.03 | 0.47 |
| **Guatemala** | 0.07 | -0.09 | 0.21 | -0.01 | -0.04 | 0.01 | 0.06 | | -0.11 | 0.20 |
| **India** | 0.22 | 0.03 | 0.41 | 0.01 | -0.03 | 0.04 | 0.23 | | 0.05 | 0.42 |
| **Pakistan** | 0.26 | 0.08 | 0.48 | 0.00 | -0.01 | 0.01 | 0.26 | | 0.08 | 0.48 |
| **Adjusted mean differences in z-scores (95% CIs) for birth weight** | | | | | | | | | | |
| **Combined** | 0.16 | 0.09 | 0.23 | 0.00 | -0.01 | 0.00 | 0.16 | | 0.09 | 0.23 |
| **DRC** | 0.17 | -0.01 | 0.34 | 0.00 | -0.01 | 0.02 | 0.17 | | -0.01 | 0.34 |
| **Guatemala** | 0.07 | -0.05 | 0.22 | -0.01 | -0.03 | 0.01 | 0.06 | | -0.05 | 0.21 |
| **India** | 0.19 | 0.02 | 0.35 | 0.01 | -0.03 | 0.02 | 0.20 | | 0.03 | 0.35 |
| **Pakistan** | 0.13 | -0.02 | 0.31 | 0.00 | -0.01 | 0.01 | 0.13 | | -0.01 | 0.31 |
| **Adjusted mean differences in z-scores (95% CIs) for birth head circumference** | | | | | | | | | | |
| **Combined** | 0.09 | 0.00 | 0.18 | -0.003 | -0.009 | 0.001 | 0.09 | | -0.004 | 0.18 |
| **DRC** | 0.02 | -0.15 | 0.19 | 0.00 | -0.02 | 0.01 | 0.02 | | -0.15 | 0.19 |
| **Guatemala** | 0.04 | -0.13 | 0.22 | -0.01 | -0.02 | 0.00 | 0.03 | | -0.14 | 0.21 |
| **India** | 0.16 | -0.02 | 0.36 | 0.01 | -0.03 | 0.05 | 0.17 | | -0.02 | 0.36 |
| **Pakistan** | 0.11 | -0.08 | 0.34 | 0.00 | -0.01 | 0.01 | 0.11 | | -0.08 | 0.34 |

DRC, Democratic Republic of Congo; WHO, World Health Organization; 95% CIs, 95% confidence intervals

The results are presented after combining Arm 1 and Arm 2 and comparing with Arm 3.

Arm 1+Arm 2 vs. Arm 3: Comparison of women who consumed a small quantity lipid-based nutrient supplement from preconception until birth (Arm 1) and women who started nutrition supplement at 12 weeks of pregnancy (Arm 2) versus women who did not receive the supplement at all (Arm 3: control Arm)

Z-scores for birth length, weight and head circumference -for-age are based on WHO child growth standards that account for newborn’s sex and age (non-gestational age adjusted data) (3).

Two way-decomposition of total effect into direct and indirect effect (mediated by hemoglobin at 12 weeks of pregnancy) was done after adjusting for M-Y confounders: Cluster, maternal age, parity, education, socioeconomic status, pre-pregnancy hemoglobin, and pre-pregnancy body mass index.

| **Supplemental Table 16: Decomposition of total effect of preconception small quantity lipid-based nutrition supplement into direct and indirect effects- Pooling site-specific results using meta-analysis after employing random effect models** | | | | | | | | | |
| --- | --- | --- | --- | --- | --- | --- | --- | --- | --- |
| **(12 weeks of gestation)** | | | | | | | | | |
| **Adjusted mean differences and 95% CIs: Arm 1 vs. Arm 3: Model 1^1^** | | | | | | | | | |
|  | **Direct effect** | | | **Indirect Effect** | | | **Total Effect** | | |
|  | **Beta-coefficient** | **lower CI** | **Upper CI** | **Beta-coefficient** | **lower CI** | **Upper CI** | **Beta-coefficient** | **lower CI** | **Upper CI** |
| **Birth length Z-scores** | 0.16 | 0.01 | 0.30 | 0.02 | -0.03 | 0.07 | 0.18 | 0.03 | 0.33 |
| **Birth weight Z-scores** | 0.10 | 0.01 | 0.23 | 0.01 | -0.04 | 0.06 | 0.12 | 0.005 | 0.23 |
| **Birth head circumference Z-scores** | 0.04 | -0.06 | 0.14 | 0.02 | -0.04 | 0.08 | 0.06 | -0.06 | 0.17 |
| **Adjusted mean differences and 95% CIs: Arm 1 vs. Arm 3: Model 2^2^** | | | | | | | | | |
| **Birth length Z-scores** | 0.15 | 0.003 | 0.30 | 0.03 | -0.03 | 0.08 | 0.18 | 0.02 | 0.33 |
| **Birth weight Z-scores** | 0.10 | -0.003 | 0.07 | 0.01 | -0.04 | 0.07 | 0.11 | 0.0001 | 0.23 |
| **Birth head circumference Z-scores** | 0.03 | -0.07 | 0.14 | 0.02 | -0.04 | 0.08 | 0.05 | -0.07 | 0.17 |
| **(32 weeks of gestation)** | | | | | | | | | |
| **Adjusted mean differences and 95% CIs: Arm 1 vs. Arm 3: Model 1^1^** | | | | | | | | | |
| **Birth length Z-scores** | 0.21 | -0.40 | 0.82 | -0.01 | -0.04 | 0.01 | 0.20 | -0.40 | 0.80 |
| **Birth weight Z-scores** | 0.18 | -0.37 | 0.74 | -0.01 | -0.03 | 0.01 | 0.17 | -0.39 | 0.73 |
| **Birth head circumference Z-scores** | 0.05 | -0.93 | 1.03 | -0.01 | -0.04 | 0.02 | 0.04 | -0.94 | 1.02 |
| **Adjusted mean differences and 95% CIs: Arm 1 vs. Arm 3: Model 2^2^** | | | | | | | | | |
| **Birth length Z-scores** | 0.21 | -0.41 | 0.84 | -0.01 | -0.03 | 0.01 | 0.20 | -0.43 | 0.83 |
| **Birth weight Z-scores** | 0.16 | -0.42 | 0.76 | -0.01 | -0.03 | 0.01 | 0.15 | -0.43 | 0.75 |
| **Birth head circumference Z-scores** | 0.04 | -0.92 | 1.01 | -0.01 | -0.03 | 0.01 | 0.03 | -0.93 | 1.00 |

95% CIs, 95% confidence intervals; Hb, hemoglobin; WHO, World Health Organization

The decomposition of the total effect of the natural direct and indirect effect was adjusted for mediator-outcome (M-Y) confounders: maternal age, parity, education, socioeconomic status, pre-pregnancy hemoglobin, and pre-pregnancy body mass index. We included both linear and quadratic terms of hemoglobin (both were centered to mean) and their interaction terms with intervention Arm.

Z-scores for birth length, weight and head circumference -for-age are based on WHO child growth standards that account for newborn’s sex and age (non-gestational age adjusted data) (3).

^1^ Model 1: We kept centered Hb in linear form and included interaction between Arm and Hb-12 (Hb_12*Arm)

^2^ Model 2: We kept centered Hb in linear form and included interaction between Arm and Hb-12 (Hb_12*Arm) and interaction term between Arm and pre-pregnancy Hb (Arm*Hb at baseline)

**Supplemental Table 17: Decomposition of total effect of preconception small quantity lipid-based nutrition supplement on markers of intrauterine growth into direct and indirect effects: Complete case analysis and multiple imputation findings**

| **12 weeks of gestation (Arm 1 vs. Arm 3)** | | | |
| --- | --- | --- | --- |
| **Adjusted mean differences and 95% CIs: Complete case analysis** | | | |
|  | **Direct effect** | **Indirect effect** | **Total effect** |
| **Birth length Z-scores** | 0.22 (0.09, 0.34) | -0.02 (-0.04, 0.01) | 0.20 (0.07, 0.32) |
| **Birth weight Z-scores** | 0.13 (0.02, 0.24) | -0.01 (-0.03, 0.01) | 0.12 (0.01, 0.23) |
| **Birth head circumference Z-scores** | 0.08 (-0.04, 0.20) | -0.01 (-0.04, 0.02) | 0.07 (-0.05, 0.20) |
| **Adjusted mean differences and 95% CIs: Multiple imputation results** | | | |
| **Birth length Z-scores** | 0.21 (0.09, 0.33) | -0.02 (-0.04, 0.01) | 0.19 (0.08, 0.30) |
| **Birth weight Z-scores** | 0.15 (0.06, 0.26) | -0.01 (-0.03, 0.01) | 0.14 (0.05, 0.24) |
| **Birth head circumference Z-scores** | 0.08 (-0.03, 0.19) | -0.01 (-0.03, 0.02) | 0.07 (-0.04, 0.17) |
| **32 weeks of gestation (Arm 1 vs. Arm 3)** | | | |
| **Adjusted mean differences and 95% CIs: Complete case analysis** | | | |
| **Birth length Z-scores** | 0.22 (0.09, 0.33) | -0.014 (-0.03, 0.001) | 0.20 (0.07, 0.32) |
| **Birth weight Z-scores** | 0.17 (0.07, 0.27) | -0.01 (-0.02, 0.001) | 0.16 (0.06, 0.26) |
| **Birth head circumference Z-scores** | 0.10 (-0.004, 0.22) | -0.01 (-0.02, 0.002) | 0.09 (-0.02, 0.21) |
| **Adjusted mean differences and 95% CIs: Multiple imputation results** | | | |
| **Birth length Z-scores** | 0.21 (0.09, 0.32) | -0.02 (-0.03, 0.002) | 0.19 (0.08, 0.30) |
| **Birth weight Z-scores** | 0.15 (0.06, 0.25) | -0.01 (-0.02, 0.001) | 0.14 (0.05, 0.24) |
| **Birth head circumference Z-scores** | 0.07 (-0.03, 0.18) | -0.01 (-0.03, 0.002) | 0.06 (-0.04, 0.17) |
| **32 weeks of gestation (Arm 2 vs. Arm 3)** | | | |
| **Adjusted mean differences and 95% CIs: Complete case analysis** | | | |
| **Birth length Z-scores** | 0.22 (0.11, 0.32) | -0.0003 (-0.003, 0.002) | 0.22 (0.11, 0.32) |
| **Birth weight Z-scores** | 0.15 (0.0.5, 0.24) | 0.0001 (-0.001, 0.001) | 0.15 (0.05, 0.24) |
| **Birth head circumference Z-scores** | 0.09 (-0.01, 0.20) | 0.001 (-0.001, 0.002) | 0.09 (-0.01, 0.20) |
| **Adjusted mean differences and 95% CIs: Multiple imputation results** | | | |
| **Birth length Z-scores** | 0.20 (0.09, 0.30) | -0.0006 (-0.005, 0.004) | 0.20 (0.09, 0.30) |
| **Birth weight Z-scores** | 0.13 (0.04, 0.22) | -0.001 (-0.001, 0.001) | 0.13 (0.04, 0.22) |
| **Birth head circumference Z-scores** | 0.06 (-0.04, 0.17) | 0.001 (-0.001, 0.002) | 0.06 (-0.03, 0.17) |

95% CIs, 95% confidence intervals; WHO, World Health Organization

Arm 1 vs. Arm 3: Comparison of women who consumed a small quantity lipid-based nutrient supplement from preconception until birth (Arm 1) vs. women who did not take the supplement at all (Arm 3: control Arm) Arm 2 vs. Arm 3: Comparison of women who consumed a small quantity lipid-based nutrient supplement from the second trimester of pregnancy until birth (Arm 2) vs. women who did not take the supplement (Arm 3: control Arm)

Z-scores for birth length, weight and head circumference -for-age are based on WHO child growth standards that account for newborn’s sex and age (non-gestational age adjusted data) (3).

Decomposition of the total effect into natural direct and indirect effect was done after keeping interaction terms (Arm* Hb during pregnancy and Arm*Pre-pregnancy Hb) and adjusting for mediator-outcome (M-Y) confounders: Site, maternal age, parity, education, socioeconomic status, pre-pregnancy hemoglobin, and pre-pregnancy body mass index.

**Supplemental Table 18: Decomposition of total effect of preconception small quantity lipid-based nutrition supplement on markers of intrauterine growth into direct and indirect effects: Analysis on sample of women with complete data on Hb both at 12 and 32 weeks of gestation (n=1861)**

| ***Arm 1 vs. Arm 3: 12 weeks of gestation*** | | | | | | | | | | | | | | | | | | | | | |
| --- | --- | --- | --- | --- | --- | --- | --- | --- | --- | --- | --- | --- | --- | --- | --- | --- | --- | --- | --- | --- | --- |
| **Markers of intrauterine growth** | **Direct effect** | | | | | | | | **Indirect Effect** | | | | | | | **Total Effect** | | | | | |
|  | **Beta-coefficient** | | | **lower CI** | | **Upper CI** | | **Beta-coefficient** | | | **lower CI** | | **Upper CI** | | **Beta-coefficient** | | | **lower CI** | | **Upper CI** |  |
| **Birth length Z-scores** | | 0.23 | 0.10 | | 0.35 | | 0.001 | | | -0.02 | | 0.02 | | 0.23 | | | 0.10 | | 0.35 | |  |
| **Birth weight Z-scores** | | 0.16 | 0.05 | | 0.28 | | 0.0005 | | | -0.02 | | 0.01 | | 0.16 | | | 0.05 | | 0.29 | |  |
| **Birth head circumference Z-scores** | | 0.12 | -0.01 | | 0.25 | | 0.001 | | | -0.02 | | 0.02 | | 0.12 | | | -0.002 | | 0.25 | |  |
| ***Arm 1 vs. Arm 3: 32 weeks of gestation*** | | | | | | | | | | | | | | | | | | | | | |
| **Birth length Z-scores** | | 0.24 | 0.1 | | 0.36 | | -0.005 | | | -0.01 | | 0.004 | | 0.23 | | | 0.1 | | 0.36 | |  |
| **Birth weight Z-scores** | | 0.16 | 0.04 | | 0.27 | | -0.003 | | | 0.01 | | 0.003 | | 0.16 | | | 0.04 | | 0.27 | |  |
| **Birth head circumference Z-scores** | | 0.13 | -0.01 | | 0.24 | | -0.01 | | | -0.01 | | 0.005 | | 0.12 | | | -0.01 | | 0.24 | |  |
| ***Arm 2 vs. Arm 3: 32 weeks of gestation*** | | | | | | | | | | | | | | | | | | | | | |
| **Birth length Z-scores** | | 0.23 | 0.11 | | 0.34 | | -0.002 | | | -0.01 | | 0.002 | | 0.23 | | | 0.11 | | 0.34 | |  |
| **Birth weight Z-scores** | | 0.16 | 0.04 | | 0.27 | | -0.003 | | | -0.01 | | 0.003 | | 0.16 | | | 0.03 | | 0.27 | |  |
| **Birth head circumference Z-scores** | | 0.12 | -0.01 | | 0.27 | | -0.003 | | | -0.01 | | 0.004 | | 0.12 | | | -0.01 | | 0.27 | |  |

CI: Confidence interval; Hb: hemoglobin; Arm 1 vs. Arm 3: Comparison of women who consumed a small quantity lipid-based nutrient supplement from preconception until birth (Arm 1) vs. women who did not take the supplement at all (Arm 3: control Arm)

Arm 2 vs. Arm 3: Comparison of women who consumed a small quantity lipid-based nutrient supplement from the second trimester of pregnancy until birth (Arm 2) vs. women who did not take the supplement (Arm 3: control Arm)

Z-scores for birth length, weight and head circumference-for-age are based on WHO child growth standards that account for newborn’s sex and age (non-gestational age adjusted data) (3).

Decomposition of the total effect into direct and indirect effect was done after adjusting for mediator-outcome (M-Y) confounders: Site, maternal age, parity, education, socioeconomic status, pre-pregnancy hemoglobin, and pre-pregnancy body mass index.

| **Supplemental Table 19: Decomposition of total effect of preconception small quantity lipid-based nutrition supplement on markers of intrauterine growth into direct and indirect effects: Subgroup analysis on women with Hb<12 g/dL and Hb≥12 g/dL** | | | | | | | | | |
| --- | --- | --- | --- | --- | --- | --- | --- | --- | --- |
| **Arm 1 vs. Arm 3: 12 weeks of gestation (Hb<12 g/dL, n=1365)** | | | | | | | | | |
| **Markers of intrauterine growth** | **Direct effect** | | | **Indirect Effect** | | | **Total Effect** | | |
|  | **Beta-coefficient** | **lower CI** | **Upper CI** | **Beta-coefficient** | **lower CI** | **Upper CI** | **Beta-coefficient** | **lower CI** | **Upper CI** |
| LAZ | 0.16 | 0.01 | 0.31 | 0.02 | -0.001 | 0.04 | 0.18 | 0.03 | 0.33 |
| WAZ | 0.06 | -0.07 | 0.19 | 0.01 | -0.01 | 0.02 | 0.07 | -0.06 | 0.20 |
| HCAZ | -0.02 | -0.17 | 0.12 | 0.01 | -0.003 | 0.03 | -0.01 | -0.15 | 0.14 |
| **Arm 1 vs. Arm 3: 12 weeks of gestation (Hb≥12 g/dL, n=710)** | | | | | | | | | |
| LAZ | 0.25 | 0.05 | 0.45 | -0.0001 | -0.006 | 0.005 | 0.25 | 0.05 | 0.45 |
| WAZ | 0.20 | 0.02 | 0.37 | -0.001 | -0.01 | 0.004 | 0.20 | 0.02 | 0.37 |
| HCAZ | 0.16 | -0.03 | 0.36 | -0.0005 | -0.02 | 0.02 | 0.16 | -0.03 | 0.36 |
| **Arm 1 vs. Arm 3: 32 weeks of gestation): (Hb<12 g/dL, n=1535)** | | | | | | | | | |
| LAZ | 0.22 | 0.08 | 0.35 | -0.0005 | -0.004 | 0.004 | 0.22 | 0.08 | 0.35 |
| WAZ | 0.16 | 0.04 | 0.28 | -0.0002 | -0.003 | 0.002 | 0.16 | 0.04 | 0.28 |
| HCAZ | 0.12 | -0.01 | 0.25 | -0.001 | -0.01 | 0.007 | 0.12 | -0.01 | 0.25 |
| **Arm 1 vs. Arm 3: 32 weeks of gestation: (Hb≥12 g/dL, n=622)** | | | | | | | | | |
| LAZ | 0.20 | 0.003 | 0.39 | 0.01 | -0.02 | 0.04 | 0.20 | 0.01 | 0.4 |
| WAZ | 0.08 | -0.08 | 0.25 | 0.005 | -0.01 | 0.02 | 0.09 | -0.08 | 0.26 |
| HCAZ | 0.02 | -0.17 | 0.22 | 0.01 | -0.01 | 0.02 | 0.03 | -0.17 | 0.23 |

CI: Confidence interval; Hb: hemoglobin; Arm 1 vs. Arm 3: Comparison of women who consumed a small quantity lipid-based nutrient supplement from preconception until birth (Arm 1) vs. women who did not take the supplement at all (Arm 3: control Arm)

Arm 2 vs. Arm 3: Comparison of women who consumed a small quantity lipid-based nutrient supplement from the second trimester of pregnancy until birth (Arm 2) vs. women who did not take the supplement (Arm 3: control Arm)

Z-scores for birth length, weight and head circumference-for-age are based on WHO child growth standards that account for newborn’s sex and age (non-gestational age adjusted data) (3).

Decomposition of the total effect into direct and indirect effect was done after adjusting for mediator-outcome (M-Y) confounders: Site, maternal age, parity, education, socioeconomic status, pre-pregnancy hemoglobin, and pre-pregnancy body mass index.

**References**

1. Waninge A, Ligthart K, Kramer J, Hoeve S, van der Schans C, Haisma H. World Health Organization, BMI classification, 2006. Measuring physical fitness.31(3):38.

2. World Health Organization. Guideline on haemoglobin cutoffs to define anaemia in individuals and populations; 2024 2024 [Available from: <https://www.guidelinecentral.com/guideline/3534081/>.

3. WHO Multicentre Growth Reference Study Group. WHO Child Growth Standards based on length/height, weight and age. Acta Paediatr Suppl. 2006;450:76-85.

4. Papageorghiou AT, Ohuma EO, Altman DG, Todros T, Cheikh Ismail L, Lambert A, et al. International standards for fetal growth based on serial ultrasound measurements: the Fetal Growth Longitudinal Study of the INTERGROWTH-21st Project. Lancet. 2014;384(9946):869-79.
